# Genetic variants associated with gout identified through a genome-wide study in the UK biobank (N = 150 542)

**DOI:** 10.1093/hmg/ddaf151

**Published:** 2025-10-11

**Authors:** Yiwen Tao, Tengda Cai, Qi Pan, Luning Yang, Sen Lin, Mainul Haque, Tania Dottorini, Abhishek Abhishek, Weihua Meng

**Affiliations:** Nottingham Ningbo China Beacons of Excellence Research and Innovation Institute, University of Nottingham Ningbo China, Ningbo 315100, China; Nottingham Ningbo China Beacons of Excellence Research and Innovation Institute, University of Nottingham Ningbo China, Ningbo 315100, China; Nottingham Ningbo China Beacons of Excellence Research and Innovation Institute, University of Nottingham Ningbo China, Ningbo 315100, China; Nottingham Ningbo China Beacons of Excellence Research and Innovation Institute, University of Nottingham Ningbo China, Ningbo 315100, China; Nottingham Ningbo China Beacons of Excellence Research and Innovation Institute, University of Nottingham Ningbo China, Ningbo 315100, China; School of Mathematical Sciences, University of Nottingham Ningbo China, Ningbo 315100, China; School of Veterinary Medicine and Science, University of Nottingham, Nottingham LE12 5RD, United Kingdom; Division of Rheumatology, Orthopaedics and Dermatology, School of Medicine, University of Nottingham, Nottingham NG5 1PB, United Kingdom; Nottingham Ningbo China Beacons of Excellence Research and Innovation Institute, University of Nottingham Ningbo China, Ningbo 315100, China; Division of Population Health and Genomics, Ninewells Hospital and Medical School, University of Dundee, Dundee DD2 4BF, United Kingdom; Center for Public Health, Faculty of Medicine, Health and Life Sciences, School of Medicine, Dentistry and Biomedical Sciences, Queen’s University Belfast, Belfast BT12 6BA, United Kingdom

**Keywords:** Gout, hyperuricemia, UK Biobank, genome-wide association, tissuespecific gene expression

## Abstract

Gout is a prevalent and painful form of inflammatory arthritis associated with hyperuricemia, which leads to monosodium urate crystal deposition in joints and surrounding tissues, triggering acute inflammatory responses. This disease is also closely linked to serious comorbidities, including cardiovascular diseases, chronic kidney diseases, diabetes, and increased mortality risk, significantly impacting global health. In this study, we conducted a comprehensive genome-wide association study (GWAS) based on the UK Biobank pain questionnaire 2019, comprising 10 474 gout cases and 140 068 controls, identifying 13 loci associated with gout. These findings were further explored in the FinnGen cohort, with 10 loci being replicated significantly. Sex-stratified analyses revealed notable differences, with 16 loci identified in males and two loci identified in females, reflecting both shared and sex -stratified genetic influences on gout susceptibility. In addition, genetic correlation analyses demonstrated strong associations between gout and traits related to urate levels, specific medication use, and metabolic functions. Transcriptome-wide association studies highlighted several genes, such as *SLC16A9* and *ASAH2B*, which showed significant expression patterns across various tissues, implicating metabolic and immune pathways in gout. Phenome-wide association studies of significant single nucleotide polymorphisms revealed links to metabolic, immunological, and skeletal traits, underscoring the multi-faceted nature of gout. These results contribute valuable insights into the genetic architecture and biological mechanisms underlying gout, suggesting potential avenues for tailored interventions.

## Introduction

Gout is a common and painful inflammatory arthritis that affects millions of people worldwide. It is commonly related to hyperuricemia, leading to the deposition of monosodium urate crystals in joints and tissues, triggering acute inflammation [1, 2]. Most instances of gout are characterized by the rapid development of severe acute monarticular arthritis in a peripheral joint of the leg [3]. Over recent decades, the incidence of gout has steadily increased, largely due to lifestyle factors such as diet, obesity, and metabolic conditions [4]. Gout is also significantly linked to cardiovascular diseases, kidney diseases, diabetes, and early mortality, further compounding its impact on public health [5, 6].

Epidemiological evidence from multiple countries indicates that gout is becoming increasingly prevalent, with men being more susceptible than women. Globally, the prevalence of gout is estimated to range from less than 1% to 6.8%, while its incidence varies between 0.58 and 2.89 per 1000 person-years, depending on the population studied and the methodologies used [7]. In 2020, an estimated 55.8 million people worldwide were living with gout reflecting a 22.5% increase in the age-standardized prevalence rate globally since 1990 [8]. In line with the global trend, China has experienced a substantial rise in gout burden, with prevalence increasing from approximately 0.64% to 0.81%, and the number of affected individuals growing from around 6 million to nearly 17 million between 1990 and 2021 [9]. Similarly, in Australia, self-reported data from the 2022 Australian National Health Survey indicated that 0.9% of adults have gout [10]. In the United States, gout incidence rose by 59.53%, prevalence increased by 71.44%, and disability-adjusted life years grew by 69.33% between 1990 and 2021 [11].

Genetic factors play a crucial role in the development of gout, with several studies highlighting its strong hereditary component. Twin studies have demonstrated a strong genetic component in gout, with heritability estimates reaching 60% for uric acid kidney clearance, 87% for uric acid-to-creatinine ratios, and 28% to 31% for gout itself [7, 12]. Additionally, a population-based study confirmed that gout runs in families, with higher risk for individuals with a family history, influenced by both genetic and environmental factors that vary by sex [13]. A study from the UK Biobank further estimated that the heritability of elevated serum urate and gout, driven by common genetic variants, is similar among European individuals [12]. Genome-wide association studies (GWAS) have greatly advanced our understanding of the genetic architecture of gout. Several genetic loci have been identified to be associated with gout risk, including *SLC2A9*, *ABCG2*, *SLC17A3, ABCG2*, *SLC2A9*, *SLC22A11*, *GCKR*, *MEPE*, *PPM1K-DT*, *LOC105377323* and *ADH1B* [14, 15]. During the preparation of this paper, a new genome-wide association analysis revealed 377 loci and 410 genetically independent signals associated with gout, including 149 previously unreported loci [16].

The purpose of this study was to identify genetic variants associated with gout using data from the UK Biobank, specifically leveraging the 2019 pain questionnaire to refine the gout phenotype definition. This updated definition resulted in a larger number of identified gout cases compared to previous studies using earlier UK Biobank data. Sex-stratified GWAS were conducted to investigate potential genetic variants with differential effects between males and females. Furthermore, we sought to replicate our findings using the FinnGen Biobank cohort [17] and performed post-GWAS analyses, including pathway analysis and gene prioritization, to gain deeper insights into the biological mechanisms driving gout.

## Results

### Sample description

The study utilized data from the UK Biobank’s 2019 pain questionnaire, which provided updated information on gout. A total of 166 733 participants completed this second questionnaire, leading to the identification of a more precisely defined gout phenotype. The data were refined to include only White-British genetic ancestry who met the QC criteria. For the primary GWAS analysis, we included 150 542 individuals and 11 166 749 single nucleotide polymorphisms (SNPs), with 10 474 classified as cases (individuals reporting gout) and 140 068 as controls (those reporting no gout). The cohort consisted of 65 358 males (of which 7907 were cases and 57 451 were controls) and 85 184 females (with 2567 cases and 82 617 controls). Table 1 provides an overview of the key demographic and clinical characteristics of both the case and control groups. Statistically significant differences were observed between the groups in variables including age, sex and urate with *p* values less than 0.001.

**Table 1:** Clinical characteristics of gout cases and controls in the UK biobank.

GWAS analysis	Covariates	Cases	Controls	*p-*value
Primary GWAS	Sex (female: male)	2567: 7907	82 617: 57451	<0.001
	Age (years)	58.2 (7.08)	55.8 (7.64)	<0.001
	Urate (umol/L)	394.8 (95.15)	296.4 (72.23)	<0.001
Female-stratified GWAS	Age (years)	58.2 (6.97)	55.4 (7.56)	<0.001
	Urate (umol/L)	320.1 (94.65)	263.6 (60.281)	<0.001
Male-stratified GWAS	Age (years)	58.2 (7.12)	56.3 (7.72)	<0.001
	Urate (umol/L)	418.8 (81.94)	343.3 (61.17)	<0.001

Total number of females: 85184; Total number of males: 65358.

Continuous covariates were presented as mean (standard deviation)

BMI represents body mass index.

^a^ ${\chi^{2}}$ test was used to test the difference of gender frequency between cases and controls and an independent t test was used for other covariates.

### GWAS results

**Figure 1:**
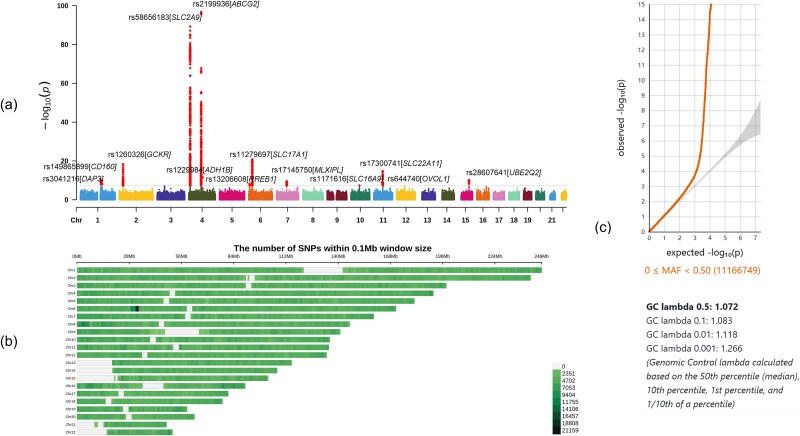
Primary GWAS analysis on gout. a) the Manhattan plot of the primary GWAS analysis on gout (N = 150 542). Each dot represents an SNP, plotted by chromosomal position along the x-axis and significance (−log10 *P* value) on the y-axis. The dashed red line indicates the cut-off *P* value of 5 × 10^−8^. b) the number of SNPs within 0.1 Mb window size on each chromosome, indicating the SNP density and highlighting regions with higher genetic variation. Chromosomes 1 and 2 exhibit the highest SNP density. **c**) the Q-Q plot of the primary GWAS, illustrating the observed versus expected p-value distribution, with GC lambda values calculated across the 50th, 10th, 1st, and 0.1 percentiles (1.072, 1.083, 1.118, and 1.266, respectively), suggesting a close fit to expectations with minor deviations at extreme values.

**Figure 2:**
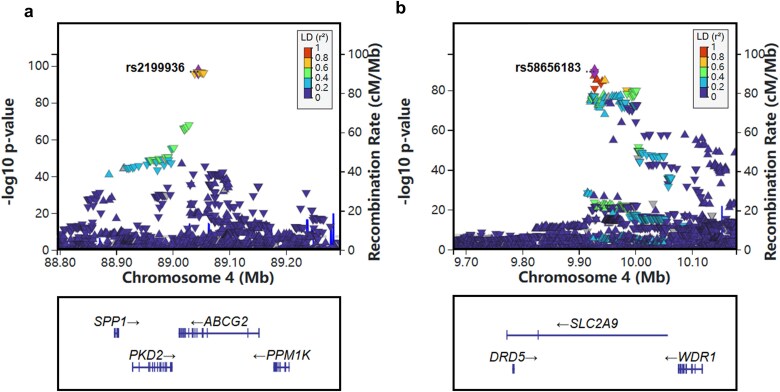
Regional plots of *ABCG2* and *SLC2A9* loci. The regional plot of loci in the *ABCG2* (a) and *SLC2A9* (b) region based on the primary GWAS association analysis. Each plot shows local SNP associations, with the lead SNP highlighted in purple. The x-axis represents the genomic position in megabases (Mb), while the y-axis displays the association strength of each SNP as –log10 *P* value.

**Figure 3:**
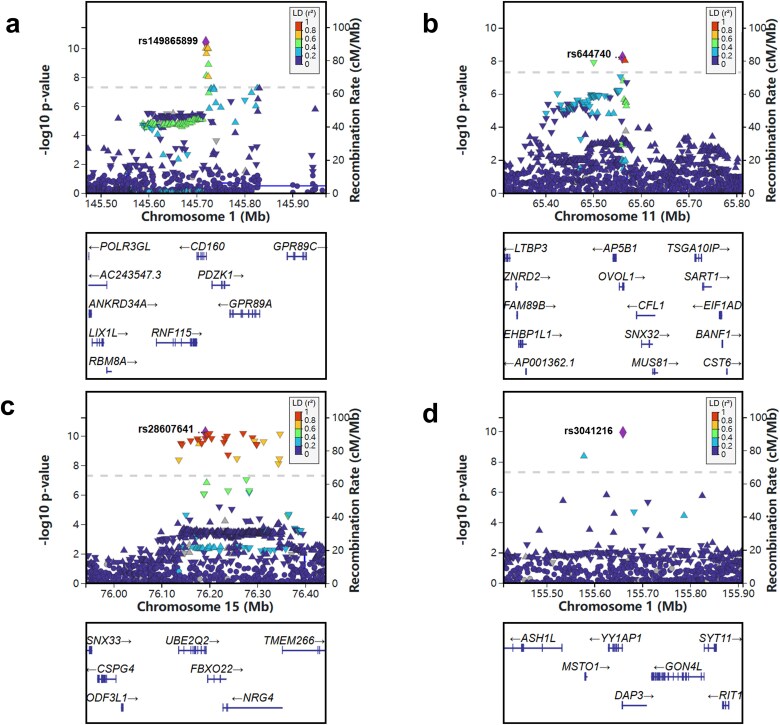
Regional plots of additional loci. The regional plots of loci in the *CD160* (a)*, OVOL1* (b)*, UBE2Q2* (c) and *DAP3* (d) regions based on the primary GWAS association analysis. Each plot shows local SNP associations, with the lead SNP highlighted in purple. The x-axis represents the genomic position in megabases (Mb), while the y-axis displays the association strength of each SNP as –log10 *p* value.

In the primary GWAS, we identified 13 loci that reached genome-wide significance (*P* < 5 × 10^−8^), as summarized in Table 2 and showed in Fig. 1a. Of these, nine loci had been previously reported, while four additional loci overlapped with findings from a recent study [16]. The most significant association was observed at rs2199936 in the *ABCG2* gene on chromosome 4, with a *p* value of 1.75 × 10^−97^. The second strongest association was at rs58656183 in *SLC2A9*, also on chromosome 4, with a *P* value of 5.52 × 10^−90^ (regional plot is provided in Fig. 2). Furthermore, we identified four additional loci, including rs149865899 in the *CD160* gene (*P* = 3.85 × 10^−11^), rs28607641 in *UBE2Q2* (*P* = 5.88 × 10^−11^), rs3041216 in *DAP3* (*P* = 1.18 x 10^−10^) and rs644740 in *OVOL1* (*P* = 6.22 x 10^−9^) (regional plots are provided in Fig. 3),which overlapped with findings from a recent study [16]. Figure 1b displays SNP density per 0.1 Mb window across chromosomes, with the highest density on chromosomes 1 and 2. The Q–Q plot for the primary GWAS is shown in Fig. 1c, with a genomic control lambda value of 1.118, indicating a close fit to expected values with slight deviations at extreme *p* values. The comprehensive list of all significant SNPs is available in Supplementary Table S1, and detailed regional plots for all significant loci are provided in Supplementary Fig. S1.

**Table 2:** The top SNPs within 13 loci identified by the GWAS on gout.

					UK Biobank discovery stage					FinnGen replication		
Locus rank	rsID	Chr	SNP position	Nearest gene	Effect allele	Non-effective allele	Frequency the effect allele	*P* value	Beta	*P* value	Beta	Identified or novel
1	rs2199936	4	89 045 331	*ABCG2*	A	G	0.1139	1.75×10^−97^	0.5095	5.01×10^−90^	0.5107	Sulem. et al
2	rs58656183	4	9 927 898	*SLC2A9*	G	A	0.2789	5.52×10^−90^	−0.3411	8.26×10^−68^	−0.3743	Sulem. et al
3	rs11279697	6	25 795 971	*SLC17A1*	ACACACC	A	0.4321	1.40×10^−21^	−0.1424	2.92×10^−11^	−0.1054	Nakayama. et al
4	rs1260326	2	27 730 940	*GCKR*	T	C	0.3937	3.75×10^−19^	0.1350	7.57×10^−17^	0.1316	Matsuo. et al
5	rs17300741	11	64 331 462	*SLC22A11*	A	G	0.4560	1.66×10^−15^	0.1183	2.49×10^−5^	0.0649	Sandoval-Plata. et al
6	rs1229984	4	100 239 319	*ADH1B*	T	C	0.0270	2.51×10^−12^	0.3122	4.21×10^−3^	0.2906	Sandoval-Plata. et al
7	rs149865899	1	145 719 488	*CD160*	CTTTTTCTTTTTTCT	C	0.4590	3.85×10^−11^	0.0994	6.94×10^−4^	0.0525	novel^a^
8	rs28607641	15	76 191 644	*UBE2Q2*	A	T	0.4825	5.88×10^−11^	−0.0971	4.36×10^−2^	−0.0310	novel^a^
9	rs3041216	1	155 659 698	*DAP3*	TCA	T	0.1318	1.18×10^−10^	−0.1505	7.90×10^−1^	−0.0058	novel^a^
10	rs17145750	7	73 026 378	*MLXIPL*	C	T	0.8410	2.55×10^−10^	0.1281	1.70×10^−4^	0.0817	Sumpter. et al
11	rs13206608	6	7 097 537	*RREB1*	A	G	0.3350	6.08×10^−9^	0.0918	1.20×10^−6^	0.0768	Sandoval-Plata. et al
12	rs644740	11	65 561 468	*OVOL1*	C	T	0.5356	6.22×10^−9^	0.0862	9.94×10^−7^	0.0764	novel^a^
13	rs1171616	10	61 468 589	*SLC16A9*	G	T	0.2307	3.90×10^−8^	−0.0968	3.27×10^−6^	−0.0983	Tin. et al

Chr: chromosome

^a^Indicates loci that were novel prior to 2024; following the publication of Major et al. (2024), these loci are no longer considered novel since they overlap with signals reported in that study.

To assess the replication of the genetic variants associated with gout identified in our primary GWAS, we examined the *p* values and effect sizes of these variants in the FinnGen M13 gout dataset. This dataset includes 12 723 gout cases and 507 487 controls. Out of the tested loci, 10 demonstrated significant replication after Bonferroni correction (*P* < 0.05/13 = 3.85 × 10^−3^), including rs2199936 in *ABCG2* on chromosome 4 (*P* = 5.01 × 10^−90^), rs58656183 in *SLC2A9* on chromosome 4 (*P* = 8.26 × 10^−68^), rs11279697 in *SLC17A1* on chromosome 6, (*P* = 2.92 × 10^−11^) and rs1260326 in *GCKR* on chromosome 2 (*P* = 7.57 × 10^−17^), rs17300741 in *SLC22A11* on chromosome 11 (*P* = 2.49 × 10^−5^), rs149865899 in *CD160* on chromosome 1 (*P* = 6.94 × 10^−4^), rs17145750 in *MLXIPL* on chromosome 7 (*P* = 1.70 × 10^−4^), rs13206608 in *RREB1* on chromosome 6 (*P* = 1.20 × 10^−6^), rs644740 in *OVOL1* on chromosome 11 (*P* = 9.94 × 10^−7^), rs1171616 in *SLC16A9* on chromosome 10 (*P* = 3.27 × 10^−6^) (Table 2).

**Figure 4:**
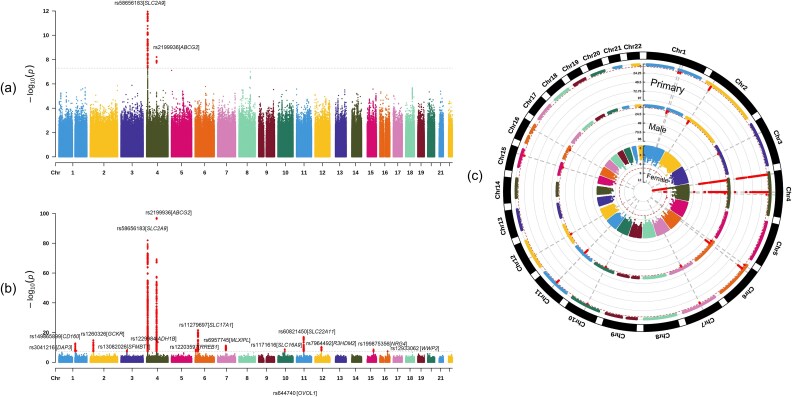
Sex-stratified GWAS analyses. a) the Manhattan plot of the female-stratified GWAS analysis (*N* = 85 184). b) the Manhattan plot of the male-stratified GWAS analysis (N = 65 358). Each dot represents an SNP, plotted by chromosomal position along the x-axis and significance (−log10 *p* value) on the y-axis. The dashed red line indicates the cut-off *p* value of 5 × 10^−8^. c) a circular Manhattan plot summarizing results from the three GWAS (primary, female- stratified, and male-stratified), providing a comprehensive comparison of significant loci across cohorts.

In sex-stratified analyses, we performed separate GWAS for males and females to examine sex-stratified genetic contribution, showed in Fig. 4. Among females, we identified two significant loci, including rs3775948 in *SLC2A9* (*p* = 1.1 × 10^−12^) and rs2199936 in *ABCG2* (*P* = 6.14 × 10^−9^), consistent with the primary analysis results (Table 3). In males, we identified 16 significant loci, with the strongest association again seen at rs74904971 in *ABCG2* (*P* = 8.52 × 10^−96^), followed by rs58656183 in *SLC2A9* (*P* = 1.06 × 10^−82^). Several loci detected in males, including those in *R3HDM2* (rs7964492, *P* = 4.31 × 10^−11^), *NRG4* (rs199875356, *P* = 2.18 × 10^−9^), *SFMBT1* (rs13082026, *P* = 9.33 x10^−9^) and *WWP2* (rs12933062, *P* = 3.54 × 10^−8^), were not genome-wide significant in the primary GWAS. The six additional GWAS on urate were provided in the Supplementary Files for reader’ interest.

### Gene, gene-set, and tissue-specific expression analysis using functional mapping and annotation (FUMA)

The gene-based analysis identified *SLC2A9* as the most significant gene linked to gout. In addition, many other genes, such as *ABCG2*, *SLC22A11*, and *SLC17A1*, also showed strong associations with gout, all surpassing the genome-wide significance threshold after the Bonferroni correction test (*P* = 0.05/19203 = 2.60 × 10^−6^). A detailed Manhattan plot is presented in Supplementary Fig. S2.

The gene-set analysis revealed the ‘GOBP_URATE_METABOLIC_PROCESS’ as the most significantly associated set (*P* = 3.28 × 10^−16^), followed by ‘GOMF_URATE_TRANSMEMBRANE_TRANSPORTER _ACTIVITY’ (*P* = 4.17 × 10^−9^) and ‘GOBP_URATE_TRANSPORT’ (*P* = 2.88 × 10^−8^). The top ten gene sets identified are listed in Supplementary Table S2.

The tissue-specific expression analysis did not yield any significant results, A summary of tissue expression levels is provided in Supplementary Fig. S3 and gene expression heatmaps are shown in Supplementary Fig. S4.

### Genetic correlation analysis using linkage disequilibrium score regression (LDSC)

The analysis revealed four correlations had ${r}_g$ values greater than 0.7, indicating a strong genetic relationship. We found a strong positive correlation with urate (${r}_g$= 0.89, *P* = 2.25 × 10^−119^). Additionally, gout showed correlations with medications including allopurinol (${r}_g$= 0.92, *P* = 4.04× 10^−82^) and colchicine (${r}_g$= 0.80, *P* = 5.43× 10^−5^). Full details are available in Supplementary Table S3. The genetic correlation between male and female participants for gout was also calculated (${r}_g$= 0.64, *P* = 5.50 × 10^−9^).

### Integration of expression quantitative trait loci (eQTL), chromatin structure, and positional mapping

The integration of eQTL analysis, chromatin interactions, and positional mapping revealed several novel insights into the genetic architecture of gout. The cis-eQTL analysis identified that the most significant association was *SNX17* expression in muscle tissue (*P* = 1.48 × 10^−88^, false discovery rates (FDR) = 2.15 × 10^−75^) on chromosome 2. The tissues most strongly associated with *ABCG2* were islets (*P* = 4.60 × 10^−12^, FDR = 3.71× 10^−10^), whole blood (*P* = 2.90 × 10^−10^, FDR = 1.46 × 10^−6^) and thyroid (*P* = 9.98 × 10^−10^, FDR = 3.34 × 10^−7^). Detailed results after Bonferroni correction are provided in Supplementary Table S4.

**Figure 5:**
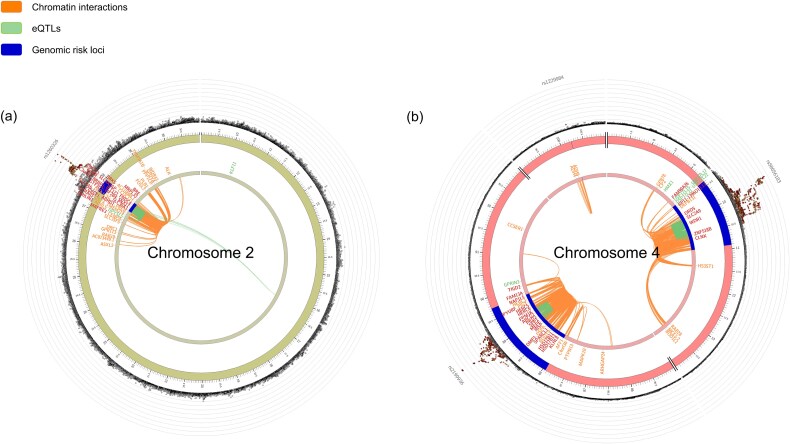
Circos plots of chromatin interactions and eQTL connections. Circos plots illustrating chromatin interactions and eQTL connections relevant to the identified gout-associated loci. The outermost layer shows a Manhattan plot with loci meeting the significance threshold of *P* < 0.05. Each SNP in a genomic risk locus is color-coded according to its maximum LD, measured by r^2^ with an independent significant SNP in the locus: Red (r^2^ > 0.8), orange (r^2^ > 0.6), green (r^2^ > 0.4), and blue (r^2^ > 0.2). The middle layer marks genomic risk loci (with lead SNP *P* < 5 × 10^−8^) in blue. The innermost layer highlights eQTLs (green) and/or chromatin interactions (orange). Plots a-b show specific top SNPs on chromosomes 2 and 4 and supplementary fig. S5 shows the other SNPs on chromosomes 1, 6, 7, 10, 11, 15 respectively.

Chromatin architecture analysis showed that on chromosome 2, *GCKR* was found to interact with genes such as *ABHD1*, *SLC30A3*, and *MRPL33*. Chromatin interactions on chromosome 4 revealed distinct regulatory regions associated with two key genes: *ABCG2* and *SLC2A9*. *ABCG2* was linked to genes like *PPM1K*, *HERC6*, and *DMP1*, while *SLC2A9* was associated with *WDR1*, *DRD5*, and *CLNK*, but no chromatin interactions were detected between *ABCG2* and *SLC2A9*. The mapped genes by chromatin interaction on the chromosomes 2 and 4 are provided in Fig. 5 and the others are provided in Supplementary Fig. S5.

**Table 3:** The top SNPs within two loci for the female-stratified GWAS and 16 loci for the male-stratified GWAS on gout.

Secondary GWAS	Locus rank	rsID	Chr	SNP position	Nearest gene	Effect allele	Non-effective allele	Frequency the effect allele	*P* value	Beta
Female- stratified GWAS	1	rs3775948	4	9 995 182	*SLC2A9*	G	C	0.2452	1.10 ×10^−12^	−0.2416
	2	rs2199936	4	89 045 331	*ABCG2*	A	G	0.1132	6.14×10^−9^	0.2684
Male- stratified GWAS	1	rs74904971	4	89 050 026	*ABCG2*	C	A	0.8852	8.52 ×10^−98^	−0.5930
	2	rs58656183	4	9 927 898	*SLC2A9*	G	A	0.2781	1.06 ×10^−82^	−0.3805
	3	rs11279697	6	25 795 971	*SLC17A1*	ACACACC	A	0.4319	2.09 ×10^−22^	−0.1691
	4	rs60821450	11	64 327 150	*SLC22A11*	C	CT	0.4379	8.04 ×10^−18^	0.1506
	5	rs1260326	2	27 730 940	*GCKR*	T	C	0.3963	1.27 ×10^−15^	0.1402
	6	rs1229984	4	100 239 319	*ADH1B*	T	C	0.0287	2.86 ×10^−14^	0.3924
	7	rs149865899	1	145 719 488	*CD160*	CTTTTTCTTTTTTCT	C	0.4608	1.80 ×10^−13^	0.1289
	8	rs6957745	7	73 056 750	*MLXIPL*	T	C	0.7985	4.54 x10^−12^	0.1494
	9	rs7964492	12	57 823 585	*R3HDM2*	A	C	0.7617	4.31 x10^−11^	0.1333
	10	rs1171616	10	61 468 589	*SLC16A9*	G	T	0.2302	1.66 x10^−9^	−0.1236
	11	rs199875356	15	76 347 375	*NRG4*	CT	C	0.4506	2.18 x10^−9^	−0.1041
	12	rs12203597	6	7 085 512	*RREB1*	G	A	0.3302	2.44 x10^−9^	0.1093
	13	rs13082026	3	52 962 681	*SFMBT1*	C	T	0.5656	9.33 x10^−9^	−0.1034
	14	rs644740	11	65 561 468	*OVOL1*	C	T	0.5359	9.37 x10^−9^	0.0992
	15	rs3041216	1	155 659 698	*DAP3*	TCA	T	0.1318	1.93 x10^−8^	−0.1527
	16	rs12933062	16	69 812 097	*WWP2*	T	A	0.6265	3.54 x10^−8^	0.1006

Chr: chromosome

### Transcriptome-wide association studies (TWAS)

Several genes demonstrated statistically significant associations across various tissues. *SLC16A9* showed significant negative associations in multiple tissues, including subcutaneous adipose (Z = −5.45, *P* = 4.97 × 10^−8^) and artery aorta (Z = −5.23, *P* = 1.65 × 10^−7^). Except for *SLC16A9*, no other significant genes from our GWAS showed tissue-specific expression associations. A detailed list of genes and their tissue-specific expression associations after Bonferroni correction can be found in Supplementary Table S5.

### Phenome-wide association studies (PheWAS)

Several SNPs showed strong associations with metabolic, immunological, and skeletal traits. The SNP rs2199936 was notably associated with uric acid levels (*P* = 1.97 × 10^−131^). rs1260326 showed a remarkable association with triglyceride and cholesterol levels (*P* = 2.29 × 10^−239^), while rs17300741 was linked to serum urate levels (*P* = 4.00 × 10^−35^), both of which are directly connected to gout pathology. SNP rs1229984 demonstrated a significant correlation with psychiatric traits, such as the number of drinks per day (*P* = 1.60 × 10^−203^). Additionally, rs644740 was significantly associated with both skeletal and metabolic traits, showing strong links to heel bone mineral density (*P* = 1.30 × 10^−23^) and uric acid levels (*P* = 2.77 × 10^−20^).

Gene-level associations also revealed critical insights. The *ABCG2* gene was highly associated with uric acid levels (*P* = 2.24 × 10^−92^), confirming its role in gout susceptibility. Similarly, *SLC2A9* was significantly linked to serum urate (*P* = 4.77 × 10^−80^) and nucleotide metabolism (*P* = 2.23 × 10^−43^). These findings reinforce the central role of urate transport and purine metabolism in gout development. The *SLC17A1* gene also displayed associations with immunological traits, such as mean corpuscular hemoglobin (*P* = 5.78 × 10^−103^). A detailed list of all traits that passed the Bonferroni correction can be found in Supplementary Table S6, with visual representations of significant phenotypes associated with these SNPs and genes provided in Supplementary Fig. S6 and S7.

### Mendelian randomization (MR) results

Mendelian randomization (MR) analyses used various phenotypes including heel bone mineral density with 426 824 participants (53 184 cases and 373 640 controls), body mass index (BMI) with 461 460 participants, and alcohol consumption with 112 117 participants [18, 19] from the IEU database (Table 4). Results from the two-sample analyses (inverse variance weighted (IVW)) and the one-sample analyses (two-stage least squares (2SLS)) are summarized in Table 5, while results from additional MR methods (MR Egger, Weighted Median, Simple Mode, and Weighted Mode) are provided in Supplementary Fig. S8 and Supplementary Table S7.

In the two-sample MR analysis, there was no evidence for a causal effect of gout on heel bone mineral density (*P* = 4.10 ×10^−1^, OR = 1.00, 95% CI: 0.99–1.02), BMI (*P* = 4.19 ×10^−1^, OR = 1.00, 95% CI: 0.98–1.01), or alcohol consumption (*P* = 9.16 ×10^−1^, OR = 1.00, 95% CI: 0.99–1.01). Conversely, genetically predicted heel bone mineral density (*P* = 9.62 ×10^−1^, OR = 1.00, 95% CI: 0.92–1.10) and alcohol consumption (*P* = 7.97 ×10^−1^, OR = 1.07, 95% CI: 0.64–1.80) showed no causal effect on gout. Reverse causality analyses only predicted that BMI was strongly associated with increased gout risk (*P* = 2.07 ×10^−6^, OR = 1.49, 95% CI: 1.27–1.76).

The one-sample MR analysis yielded largely consistent results. Gout showed no significant effect on heel bone mineral density (*P* = 2.68 x10^−1^, OR = 1.02, 95% CI: 0.98–1.06), BMI (*P* = 7.30 × 10^−1^, OR = 1.21, 95% CI: 0.41–3.58), or alcohol consumption (*P* = 1.80 x10^−1^, OR = 0.87, 95% CI: 0.70–1.07). Heel bone mineral density exhibited a modest positive effect on gout risk (*P* = 3.41 ×10^−2^, OR = 1.13, 95% CI: 1.01–1.26, while BMI again showed a significant causal effect on gout (*P* = 4.22 × 10^−5^, OR = 1.01, 95% CI: 1.00–1.01). Alcohol consumption did not show significant bidirectional causal effects (*P* = 7.58 ×10^−2^, OR = 0.87, 95% CI: 0.75–1.01).

### Protein–protein interaction (PPI) and association of potential drug targets

13 significant loci identified in the primary GWAS were used to explore their interactions. The resulting network, shown in Supplementary Fig. S9, revealed key connections between the proteins encoded by the identified loci. We further analyzed the associations between these significant genes and approved drugs, finding that *ABCG2*, *SLC22A11*, *ADH1B*, and *GCKR* are all classified as Tier-1 druggable genes. These genes interact with various approved medications, including antineoplastic agents, antihypertensive agents, uricosuric agents, and lipid-lowering agents, as detailed in Supplementary Table S8.

**Table 4:** Comprehensive details of IEU GWAS datasets for MR analyses.

GWAS dataset	ID	Sample size	Cases	Controls
Heel bone mineral density	ebi-a-GCST006979	426 824	53 184	373 640
Body mass index	ukb-b-19 953	461 460	NA	NA
Alcohol consumption	ieu-a-1283	112 117	NA	NA

**Table 5:** Results of two-sample and one-sample Mendelian randomization analyses.

Method	Exposure	Outcome	Beta	SE	P value	OR	95% CI
Two-sample (Inverse variance weighted)	Gout	Heel bone mineral density	0.0046	0.0056	4.10 ×10^−1^	1.00	0.99–1.02
	Heel bone mineral density	Gout	0.0022	0.0464	9.62 ×10^−1^	1.00	0.92–1.10
	Gout	Body mass index	−0.0047	0.0058	4.19 ×10^−1^	1.00	0.98–1.01
	Body mass index	Gout	0.4006	0.0844	2.07 ×10^−6^	1.49	1.27–1.76
	Gout	Alcohol consumption	−0.0004	0.0034	9.16 ×10^−1^	1.00	0.99–1.01
	Alcohol consumption	Gout	0.0683	0.2649	7.97 ×10^−1^	1.07	0.64–1.80
One-sample	Gout	Heel bone mineral density	0.0220	0.0199	2.68 ×10^−1^	1.02	0.98–1.06
	Heel bone mineral density	Gout	0.1188	0.0560	3.41 ×10^−2^	1.13	1.01–1.26
	Gout	Body mass index	0.1913	0.5534	7.30 ×10^−1^	1.21	0.41–3.58
	Body mass index	Gout	0.0058	0.0014	4.22 ×10^−5^	1.01	1.00–1.01
	Gout	Alcohol consumption	−0.1425	0.1063	1.80 ×10^−1^	0.87	0.70–1.07
	Alcohol consumption	Gout	−0.1399	0.0788	7.58 ×10^−2^	0.87	0.75–1.01

## Discussion

In this study, we identified 13 significant genetic loci associated with gout through the primary GWAS using the UK Biobank pain questionnaire.

The most significant association was identified at the *ABCG2* locus on chromosome 4 (rs2199936, *p* = 1.75 × 10^−97^). The strong signal at this locus reinforces the importance of *ABCG2* in the pathophysiology of gout again, as it encodes a transporter protein that plays a key role in the uric acid efflux transport [20]. Dysfunctional variants of *ABCG2* can lead to reduced uric acid excretion, thereby contributing to hyperuricemia, a major risk factor for gout [21]. Another well-established locus identified in our study was *SLC2A9* on chromosome 4 (rs58656183, *P* = 5.52 × 10^−90^). *SLC2A9* encodes a glucose transporter that also facilitates urate reabsorption in the kidney [22]. Variants in this gene have been consistently associated with serum urate levels and gout risk [5, 23]. In recent years, both *ABCG2* and *SLC2A9* have been attempted to use as drug targets to treat hyperuricemia and gout [24, 25].

Among the other genes significantly associated with gout, seven loci have also been previously identified in GWAS, including those in the genes *SLC17A1*, *GCKR*, *SLC22A11*, *ADH1B*, *MLXIPL, RREB1* and *SLC16A9* [16]. Research has shown that SNPs in the *SLC17A1* gene decrease uric acid excretion and elevate blood UA levels, contributing to hyperuricemia [26]. *GCKR* regulates glucose and lipid metabolism, helping to maintain metabolic balance and protect the liver from damage associated with excess substrates [27]. *SLC22A11* functions in the reabsorption of uric acid at the renal level, directly influencing urate homeostasis [28]. *ADH1B* is primarily known for encoding alcohol dehydrogenase enzyme, which is involved in alcohol metabolism [29]. Alcohol consumption is a well-known risk factor for gout, and *ADH1B* plays a critical role in modulating this risk. *MLXIPL* (also known as ChREBP) is associated with elevated plasma triglyceride levels, increased concentrations of liver enzymes, and a higher risk of coronary artery disease [30]. *RREB1* is a transcription factor that regulates genes involved in cellular growth and differentiation [31]. *SLC16A9* encodes a monocarboxylate transporter involved in uric acid excretion and has been shown to affect serum uric acid levels [28]. These genes contribute to gout either by directly influencing blood urate levels or by altering metabolic pathways that indirectly impact gout risk.

Among the loci, *CD160* (rs149865899, *P* = 3.85 × 10^−11^) on chromosome 1 was particularly intriguing. *CD160* is a transmembrane glycoprotein anchored by glycosylphosphatidylinositol, which is primarily known for its role in immune regulation [32]. *CD160* serves as a negative regulator of CD4+ T cell activation by interacting with herpesvirus entry mediator, thereby inhibiting T cell proliferation and cytokine production [33]. Inhibiting unnecessary immune responses while maintaining general immunity can prevent excessive immune reactions and help preserve the balance of the immune system [34]. Its association with gout suggests a potential link between immune response and the development of gout, which could offer new avenues for research into the inflammatory processes underlying the disease. As gout is characterized by acute inflammatory responses to urate crystal deposition, genetic variants in immune-related genes may play a role in determining an individual’s susceptibility to these inflammatory episodes [35, 36].

Another notable finding was the association of *UBE2Q2* (rs28607641, *P* = 5.88 × 10^−11^) on chromosome 15. This gene encodes a ubiquitin-conjugating enzyme (E2), which is involved in protein degradation pathways [37]. The ubiquitination process is essential for proteome regulation, with E2 mediating the transfer of ubiquitin-like proteins from ubiquitin-activating enzyme to target proteins [38]. Ubiquitination plays a critical role in regulating the NF-κB family of transcription factors, which are central to inflammation and carcinogenesis [39]. In the context of gout, it is possible that variants in *UBE2Q2* could affect inflammatory signaling or the degradation of proteins involved in urate metabolism. Urate crystals deposited in joints can trigger a strong inflammatory response, and altered protein degradation might exacerbate or modulate this process [40]. Future research could focus on understanding how *UBE2Q2* variants influence the balance between pro-inflammatory and anti-inflammatory signals in gout flares.

The *DAP3* gene (rs3041216, *P* = 1.18 × 10^−10^) on chromosome 1 encodes a protein that is part of the small subunit of the mitochondrial ribosome, playing a crucial role in intramitochondrial protein synthesis and apoptotic signaling [41, 42]. Previous studies indicate that mitochondria play a crucial role in triggering gout attacks, and further research could clarify the effect of this gene on mitochondria and cause gout [43]. Moreover, The dominant negative form of *DAP3* inhibits apoptosis of FAS/APO-1 and TNF-α, the latter of which binds to TNF-r1 (p55R), activates NF-κB and AP-1, and promotes inflammatory gene expression [44]*.* The identification of *DAP3* as a gout-associated gene suggests that mitochondrial health and apoptotic regulation are important factors in gout susceptibility and may represent potential targets for therapeutic intervention.

The fourth locus additionally identified in our analysis was *OVOL1* (rs644740, *P* = 6.22 × 10^−9^) on chromosome 11. *OVOL1* is an essential transcription factors for epithelial lineages in vertebrate embryonic development, playing key roles in maintaining the epithelial state and facilitating terminal differentiation during tissue homeostasis [45]. *OVOL1* has been previously associated with other complex traits, such as atopic dermatitis and metabolic conditions [46].

Our sex-stratified GWAS of gout revealed two distinct genetic loci associated with gout risk in females and 16 loci in males, suggesting potential sex-stratified genetic mechanisms contributing to the disease. In the female-stratified GWAS, the two loci (*SLC2A9* and *ABCG2*) are shared with the primary GWAS results. They do not represent novel findings in the female-stratified analysis but reaffirm the importance of these genes in regulating urate levels and gout risk across both sexes. In the male-stratified GWAS, several loci were uniquely associated with gout in males. These include *R3HDM2* (rs7964492, *P* = 4.31 ×10^−11^), *NRG4* (rs199875356, *P* = 2.18 ×10^−9^), *SFMBT1* (rs13082026, *P* = 9.33 ×10^−9^) and *WWP2* (rs12933062, *P* = 3.54 ×10^−8^). However, these genes have not been directly associated with gout, and further research is needed to explore their potential roles in gout development. One major factor of the difference in the loci number of sex-stratified GWAS is that men (7907) have a much larger number of cases than women (2567). As shown in the power estimation (Fig. S10), the power for females was slightly lower (13.0%) than for males (97.8%), further underscoring the need for larger female cohorts. Another important factor is that men (average 418.8 umol/L in this study) have naturally higher serum urate levels, a key risk factor for gout, than women (average 320.1 umol/L in this study) (Table 1). This may be because estrogen promotes the excretion of uric acid in the urine of young women [47]. Gout also differs between sexes in terms of disease presentation. Men are more likely to develop gout earlier and experience more severe symptoms, which may reflect a higher genetic burden detectable in male-stratified GWAS [48]. In contrast, women tend to develop gout later in life, and other age-related factors like comorbidities or medications add complexity to explain the sex-stratified genetic influence [48]. The 64% genetic correlation between men and women also helps explain the differences in their genetic mechanisms.

The TWAS analysis revealed several important tissue-specific gene expression associations that may contribute to the biological mechanisms underlying gout. The significant associations of *SLC16A9* expression in adipose and arterial tissues suggest a potential link to adiposity, a known risk factor for gout. Increased body weight and adiposity are important contributors to gout risk, while weight loss has been shown to have a protective effect against its development [49]. The TWAS results suggest that the genetic architecture of gout is shaped by a complex interplay of metabolic, neurological, and systemic regulatory mechanisms, with several genes showing tissue-specific influences that could be key to developing a more comprehensive understanding of gout pathogenesis.

The PheWAS analysis of gout-associated genetic variants revealed important connections between metabolic, immunological, and skeletal traits. The strong association of SNP rs2199936 and rs17300741 with uric acid levels confirms their pivotal role in urate metabolism, which is a key factor in gout development. SNPs such as rs149865899 and rs3041216 were linked to variations in white blood cell counts, suggesting that immune system function may interact with metabolic pathways to influence gout risk. These findings raise the possibility that inflammation and immune response could play a more significant role in gout than previously understood. The identification of rs1229984 was associated with alcohol consumption traits, providing insights into how genetic predispositions to alcohol metabolism influence the likelihood of developing gout. This dual influence could offer new perspectives on how skeletal traits interact with the metabolic processes leading to gout, particularly in the context of bone health. At the gene level, the strong associations between *ABCG2* and *SLC2A9* with uric acid and serum urate levels reinforce the critical role of these genes in urate transport and metabolism. *SLC2A9*’s additional associations with purine metabolism further highlight that neurological dysfunctions and gout may be linked to specific biochemical abnormalities in purine metabolism [50].

Our MR analyses consistently support BMI as a major causal determinant of gout. In the two-sample analysis, genetically predicted BMI was strongly associated with an increased risk of gout, and this finding was confirmed in the one-sample analysis using individual-level UK Biobank data with the 2SLS method. This observation aligns with existing literature that has associated higher BMI with a greater risk of developing gout [51]. Conversely, gout showed no significant causal effect on BMI in either analysis, suggesting that the observed association between gout and body weight is more likely explained by BMI predisposing to gout rather than the reverse. Heel bone mineral density revealed no causal effects in the two-sample analysis, but a modest positive association with gout was observed in the one-sample MR. Given the small effect size and lack of replication, this result should be interpreted with caution and requires validation in independent cohorts. For alcohol consumption, neither two-sample nor one-sample MR provided evidence of a direct causal effect on gout risk. While observational studies have often linked alcohol intake with gout, our MR analysis suggests that this association may be confounded by other factors or may not represent a direct causal relationship. In summary, these findings highlight the utility of MR in disentangling potential causal relationships between gout and associated traits. Future studies could further investigate the biological mechanisms linking gout with BMI and explore additional phenotypes to fully understand the systemic impact of gout.

Among the identified loci, *ABCG2*, *SLC22A11*, *ADH1B*, and *GCKR* were classified as Tier-1 druggable genes. *GCKR* was found to interact with allopurinol and febuxostat, both approved for the treatment of gout, highlighting its direct relevance to gout pathogenesis and therapy. While no other identified genes were currently linked to approved gout medications, the associated drugs targeting genes such as *ABCG2*, *SLC22A11*, and *ADH1B*—involved in metabolic and inflammatory pathways—may hold potential for repurposing in gout treatment. These findings underscore the need for further investigation to explore the therapeutic applications of these genes in gout and related conditions.

The comparison with the previous study by Major and colleagues indicates both methodological and result differences [16]. Our analysis was based on the 2019 UK Biobank pain questionnaire, whereas Major et al. used baseline data collected between 2006 and 2010, leading to natural variations in case numbers and phenotype definitions. Accordingly, our cohort included 10 474 gout cases and 140 068 controls, compared with 7131 cases and 325 239 controls in Major et al.’s study. As a result, our analysis identified 13 genome-wide significant loci, while their larger meta-analysis reported 377 loci. At several overlapping loci, the lead SNPs identified in our study differ from those reported by Major et al., although they likely reflect the same underlying association signal. Such differences in lead variants may arise from variations in phenotype definitions, sample sizes, or analytical strategies, all of which can influence which SNP emerges as the most significant marker within a locus. These observations suggest that our findings complement those of Major et al., providing additional insights by leveraging updated cohorts and refined phenotyping strategies.

Although the GWAS on gout in the UK Biobank has identified significant loci associated with the disease, several limitations must be acknowledged. One of the primary limitations relates to the disparity in the number of male and female participants. Our study included 7907 male cases compared to 2567 female cases. The smaller sample size for females reduces the statistical power to identify associations, meaning that some loci relevant to female gout may have been missed. Another limitation is the reliance on self-reported data for the classification of gout cases and controls within the UK Biobank. Self-reported diagnoses may introduce biases such as recall inaccuracies or misclassification, particularly for gout, where the condition might be underreported or confused with other joint-related issues. Furthermore, the UK Biobank data lacks detailed clinical information, such as the severity or recurrence of gout episodes, which could provide a more nuanced understanding of the genetic factors influencing different forms of the disease. Additionally, the study’s findings are derived from a predominantly European ancestry cohort, which limits the generalizability of the results to other populations. Genetic variants identified in this study may not be applicable to individuals of non-European descent, thus reducing the external validity of the findings. Future studies should aim to include more diverse populations to better understand the genetic architecture of gout across different ethnicities.

In summary, our primary GWAS identified 13 genetic loci associated with gout, including four loci that were unreported when our study was conducted but appeared in contemporaneous publications. The sex-stratified GWAS further revealed 16 loci identified in males and two loci identified in females. These findings deepen our understanding of the genetic underpinnings of gout and underscore the importance of considering sex differences in genetic studies, which may help to refine risk assessments and therapeutic strategies for gout in the future.

## Materials and methods

### Data information

This study used UK Biobank data, a large-scale health resource comprising over 500 000 participants aged 40–69 from across the UK. For this analysis, we used the pain questionnaire conducted in 2019 (https://biobank.ctsu.ox.ac.uk/crystal/ukb/docs/pain_questionnaire.pdf), with responses from 166 733 individuals. Participants provided informed consent to complete health-related questionnaires and donate biological samples. To minimize population stratification, the analysis in this study was conducted exclusively on white British participants (Field ID: 21000). Ethical approval for the UK Biobank was granted by the National Research Ethics Service (reference 11/NW/0382).

### Case and control information

We used responses to the question from the UK Biobank’s 2019 pain questionnaire: ‘Have you ever been told by a doctor that you have had any of the following conditions?’. Participants who answered ‘yes’ for gout were categorized as cases, while controls were those who answered ‘no’. This allowed us to capture self-reported gout cases and define controls with no history of gout. To ensure data quality, we excluded participants who chose ‘Do not know’ or ‘Prefer not to answer,’ as well as those with missing responses.

### GWAS design and statistical analysis

**Figure 6:**
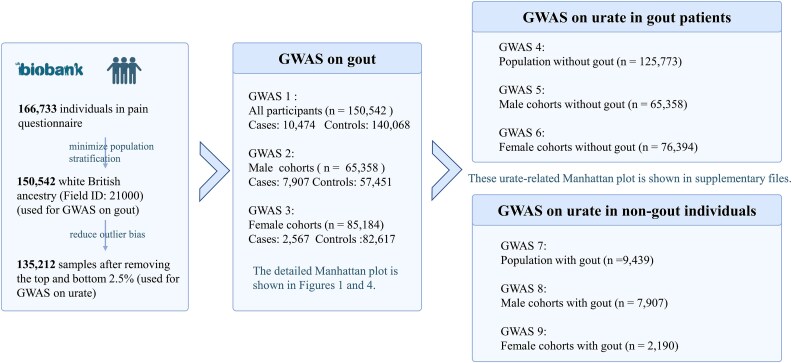
Overview of GWAS analyses on gout and urate levels. This figure summarizes the GWAS design, including 166 733 UK biobank participants, with 150 542 white British individuals selected to minimize population stratification. GWAS on gout included all participants (n = 150 542), male cohorts (n = 65 358), and female cohorts (*n* = 85 184). To reduce outlier bias, 135 212 samples were analyzed for urate GWAS after excluding the top and bottom 2.5% of urate levels, stratified into populations without gout (n = 125 773) and with gout (*n* = 9439). Detailed Manhattan plots are shown in Figs 1 and 4, with supplementary plots in supplementary files.

One primary GWAS was conducted to identify genetic variants associated with gout and two sex-stratified GWAS was performed to explore sex impact on gouts. In the replication phase, we used publicly accessible M13 gout statistics from the FinnGen dataset. This dataset includes 12 723 gout cases and 507 487 controls. For readers’ interest, we have also included six additional GWAS on urate values, details of which were described in the Supplementary Files. Figure 6 outlines the workflow of the GWAS in this study, including the three GWAS presented in the main text and six additional analyses provided in the Supplementary Files.

We used the fastGWA tool in GCTA (v1.94.1) to perform a genome-wide association analysis, applying a generalized mixed linear model [52]. Quality control measures included removing SNPs with minor allele frequencies below 0.5%, low INFO scores (< 0.3), and those violating Hardy–Weinberg equilibrium (*P* < 1 × 10^−6^). Variants on mitochondrial DNA and sex chromosomes were excluded. The analysis was adjusted for age, sex, and the first ten principal components to account for population structure. A genome-wide significance threshold of *P* < 5 × 10^−8^ was used, and narrow-sense heritability was estimated using GCTA.

We evaluated the statistical power of the primary and sex-stratified GWAS for gout using the CaTS power calculator (http://www.sph.umich.edu/csg/abecasis/CaTS/). A genome-wide significance threshold of 5 × 10^−8^, a disease allele frequency of 0.25, and a genotype relative risk of 1.15 were assumed. Disease prevalence estimates for the UK population were taken from Kuo et al., with an overall prevalence of 2.49%, 3.97% in males, and 1.05% in females [53].

### Functional annotation and genetic correlation analysis

We used FUMA to annotate the GWAS results [54]. FUMA integrates several analytical tools, including gene-based association analysis, gene-set analysis, and tissue expression analysis, using default parameters. Gene-based and gene-set analyses were conducted with MAGMA (v1.06) integrated in FUMA. In gene-based association analysis, genes were mapped to 19 023 protein-coding regions, with significance defined at *P* = 0.05/19203 = 2.60 × 10^−6^. In gene-set analysis, FUMA examines gene collections with shared biological functions, using a significance threshold of *P* = 0.05/15485 = 3.23 × 10^−6^. Tissue expression analysis was carried out using GTEx v8 datasets, which includes 54 tissue types, to identify genes with tissue-specific expression patterns. We also performed genetic correlation analysis using LDSC to estimate the genetic correlations between gout and other traits in the Complex-Traits Genetics Virtual Lab [55].

### Integration of eQTL, chromatin architecture, and positional mapping

To explore the regulatory mechanisms linked to the variants identified in the GWAS, we integrated eQTL analysis, chromatin interaction data, and positional mapping. Cis-eQTLs, which impact gene expression by interacting with nearby variants within a 1 Mb range, were the primary focus. We applied positional mapping with a 10 kb distance threshold to pinpoint regulatory elements associated with identified SNPs.

### TWAS and PheWAS

TWAS were performed to assess the impact of genetic variants on gene expression in gout-relevant tissues, utilizing GTEx v7 data [56]. PheWAS were conducted using GWAS summary statistics from 4756 GWAS results available on the ATLAS platform [57], which aimed to uncover associations between gout-linked variants and other traits, with a focus on metabolic and inflammatory phenotypes. Multiple comparisons were corrected using the Bonferroni method.

### MR analysis

We employed a two-sample MR approach to investigate the potential causal links between other traits and gout. Based on the results from our genetic correlation and PheWAS analyses, we examined whether specific genetic variants had a direct effect on gout and also investigated the reverse effect of gout on these traits. Data for the MR analysis were sourced from the UK Biobank and the IEU Open GWAS platform. The IVW method was used for the primary analysis, with additional sensitivity analyses conducted using MR Egger, Weighted Median and Simple mode approaches. Heterogeneity was assessed using Cochran’s Q test, and horizontal pleiotropy was evaluated with the MR Egger intercept test. To further validate our findings and reduce potential bias from sample overlap, we also performed one-sample MR using individual-level data from the UK Biobank via the ivmodel function in the OneSample R package. In this framework, causal estimates were obtained using a 2SLS approach, where genetic variants served as instruments for the exposure and predicted values from the first-stage regression were then regressed on the outcome in the second stage.

### P‌PI analysis and evaluation of druggability

To investigate the therapeutic potential of significant loci identified in the primary GWAS, we conducted a PPI analysis to explore interactions between these loci. The PPI analysis was performed using the STRING database (https://string-db.org). We further assessed the druggability of the candidate genes by referencing the Druggable Genome database (https://dgidb.org/)and the ChEMBL database (https://www.ebi.ac.uk/chembl). Druggable genes were categorized into three tiers: tier 1 included targets of approved drugs and clinical-phase drug candidates, tier 2 consisted of genes closely related to approved drug targets or drug-like compounds, and tier 3 comprised genes with more distant similarities to approved drug targets. Information about associated compounds, drug approval status, and therapeutic indications was retrieved from the Finan’s study on 4479 druggable genes [58] and ChEMBL database.

## Supplementary Material

Supplemental_Figures_ddaf151

Supplementary_Table_1_ddaf151

Supplementary_Table_2_ddaf151

Supplementary_Table_3_ddaf151

Supplementary_Table_4_ddaf151

Supplementary_Table_5_ddaf151

Supplementary_Table_6_ddaf151

Supplementary_Table_7_ddaf151

Supplementary_Table_8_ddaf151

Supplementary_Files_ddaf151

## Data Availability

The summary statistics of the UK Biobank results on gout are available at https://figshare.com/articles/ataset/GWAS_on_gout/30146044.
