# Supplementary material for: Genetic variants associated with gout identified through a genome-wide study in the UK biobank (N = 150 542)
Source: Hum Mol Genet. 2025 Oct 11;34(23):1951–63. doi: 10.1093/hmg/ddaf151 (PMC12627942; doi:10.1093/hmg/ddaf151)
Supplement: Supplemental_Figures_ddaf151 [file supplemental_figures_ddaf151.docx]

**Novel genetic variants associated with gout identified through a genome-wide study in the UK Biobank (N = 150,542)**

Yiwen Tao^1^, Tengda Cai^1^, Qi Pan^1^, Luning Yang^1^, Sen Lin^1^, Mainul Haque^2^, Tania Dottorini^3^, Abhishek Abhishek^4^, Weihua Meng^1,5,6^ *.

**
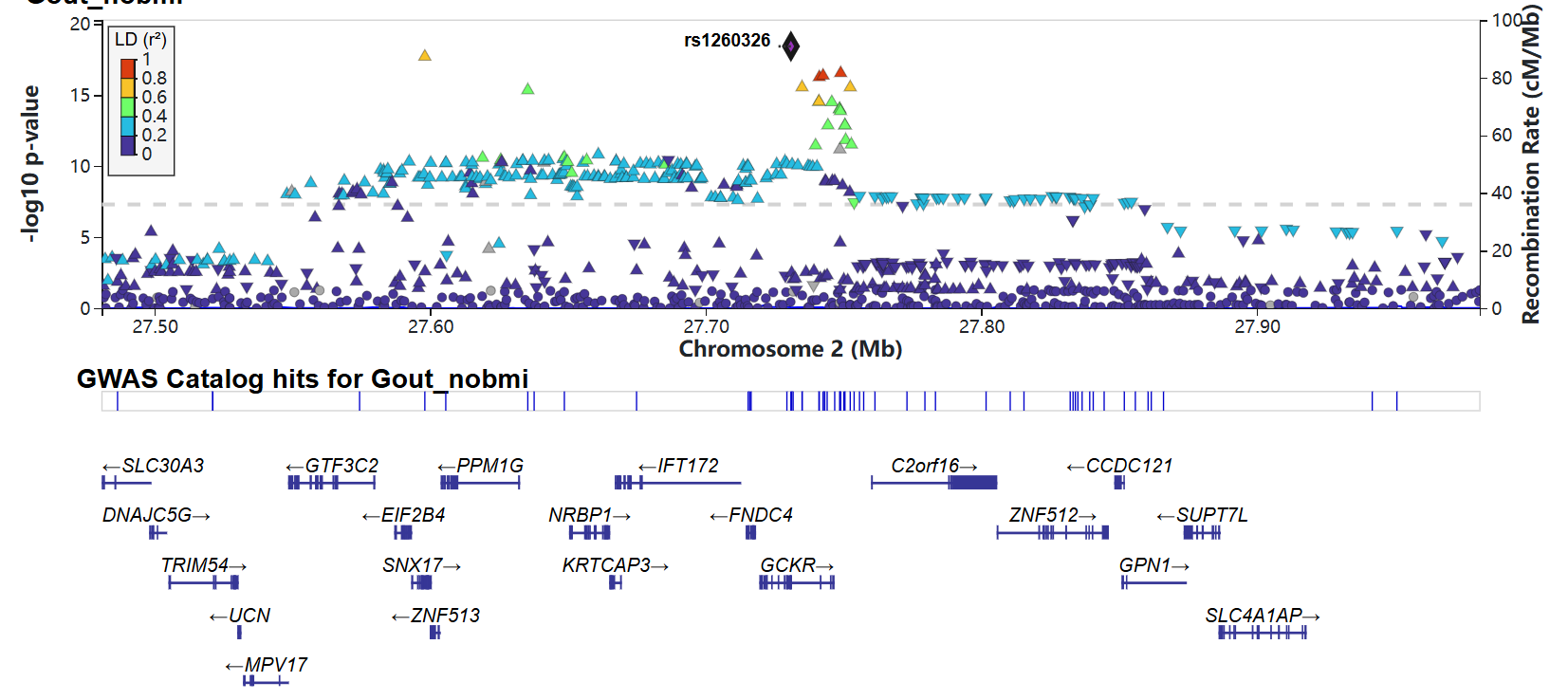
Supplementary Figures and Tables**

B

A

**
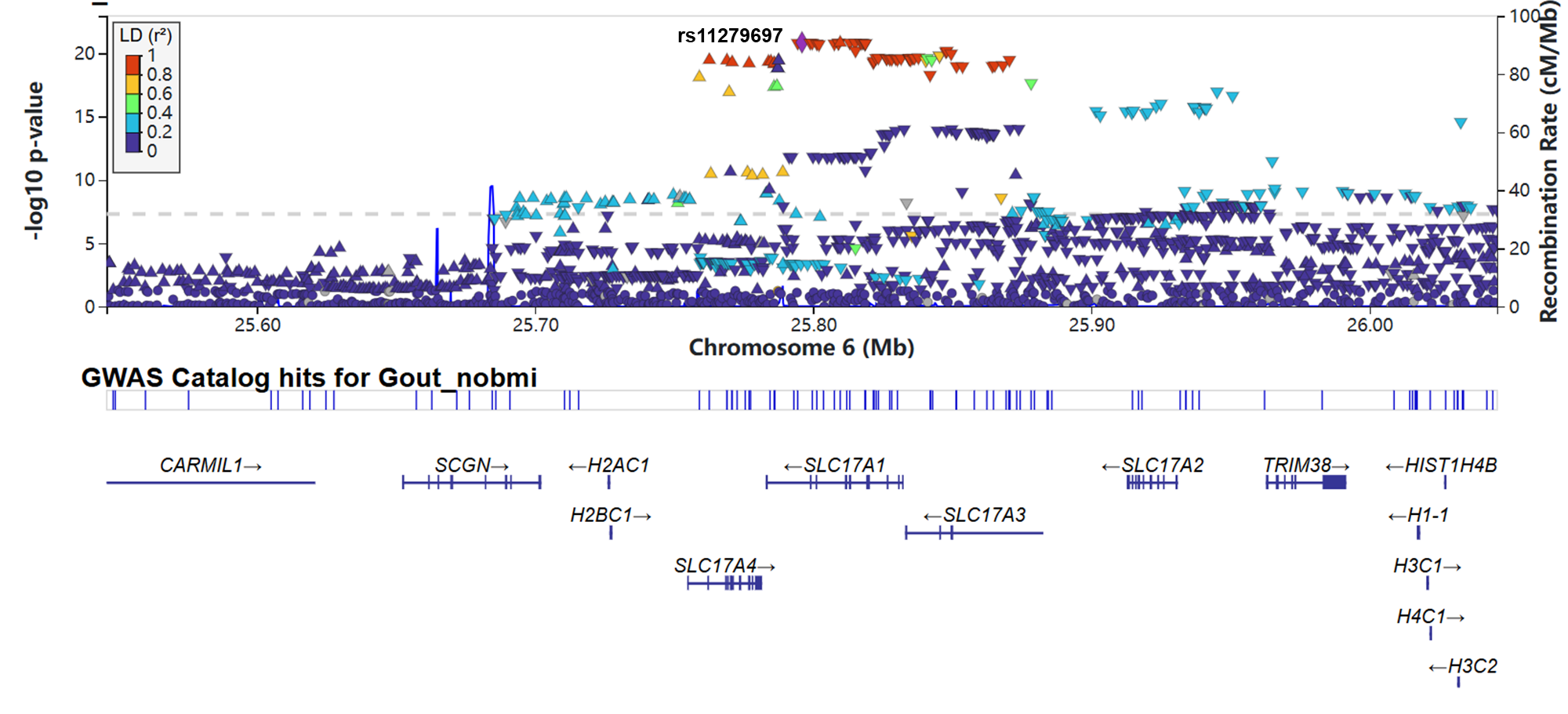

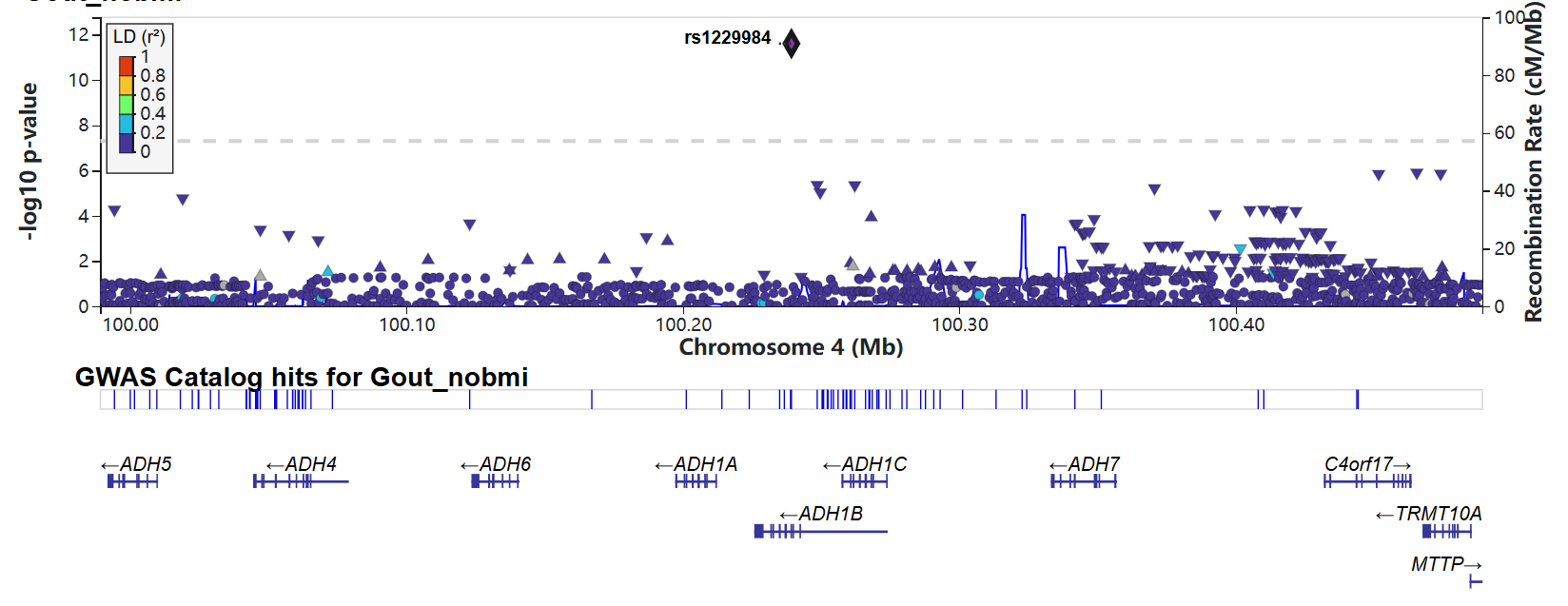
**

**
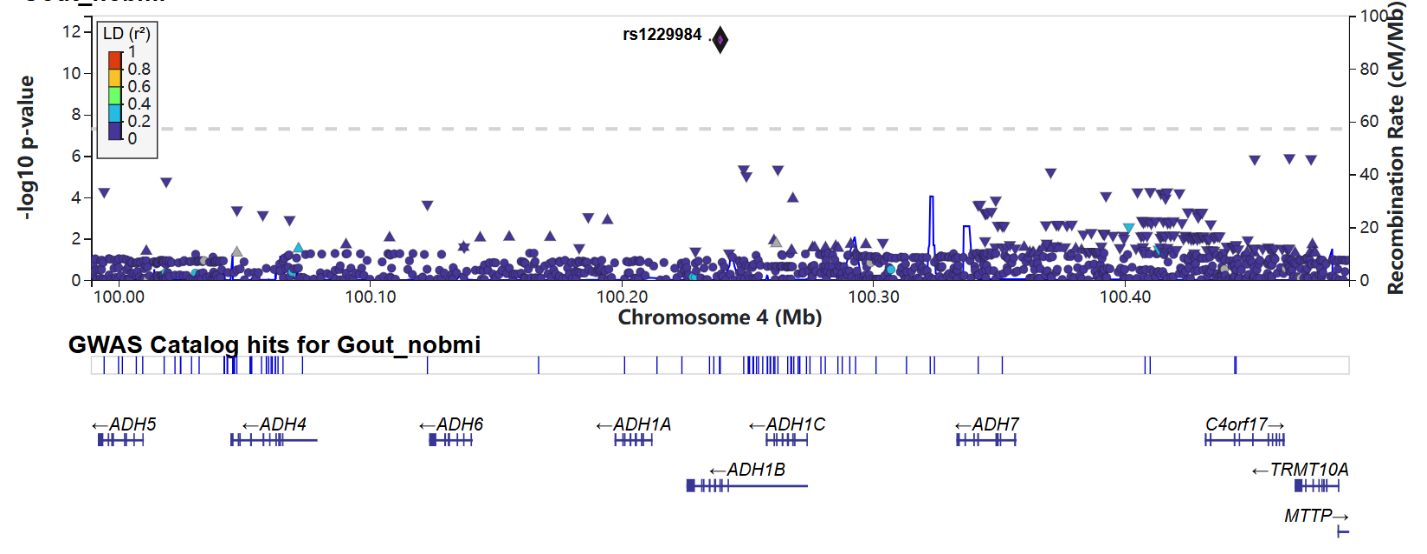

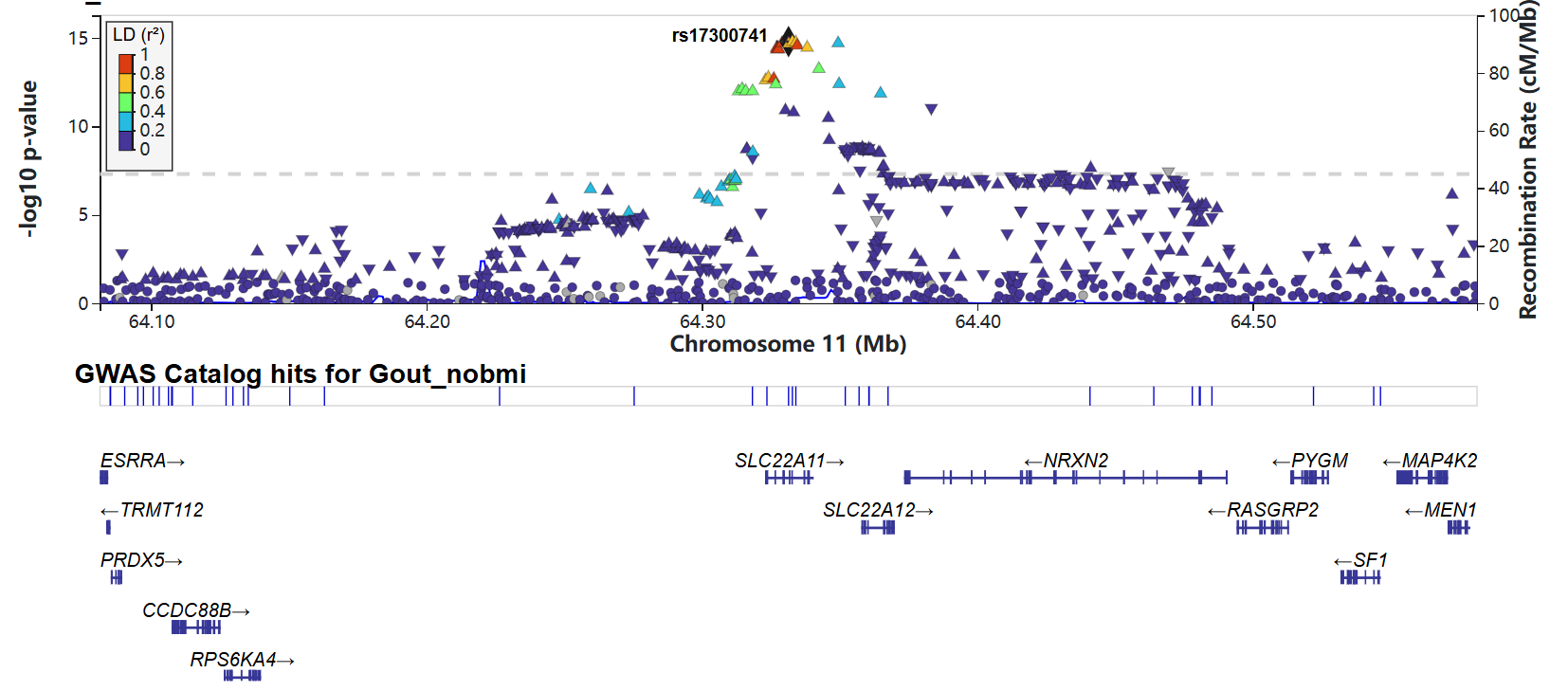
**

E

D

C

**
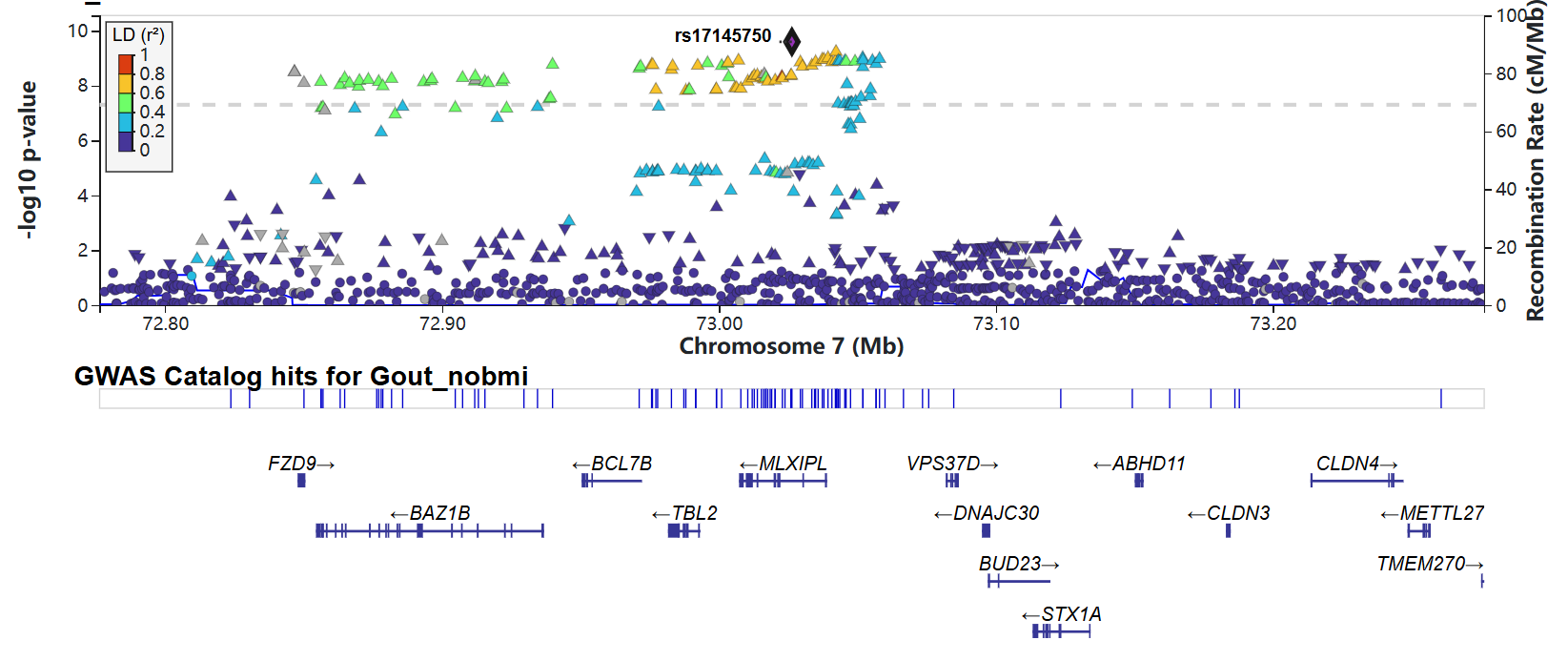
**

**
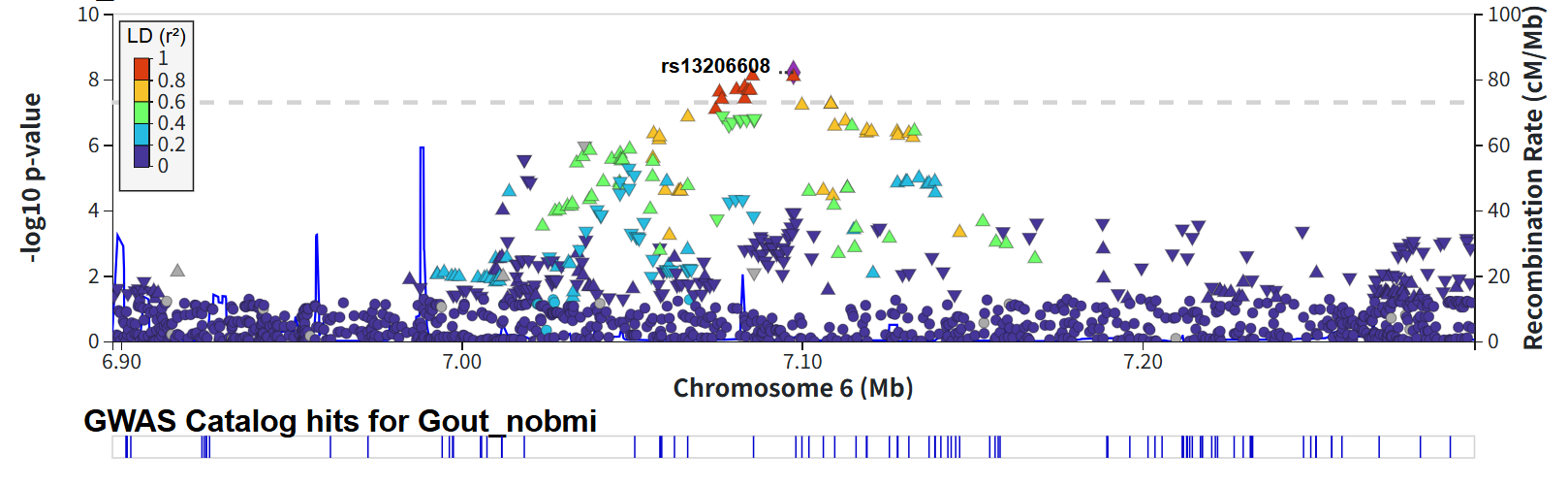
**

G

F

**
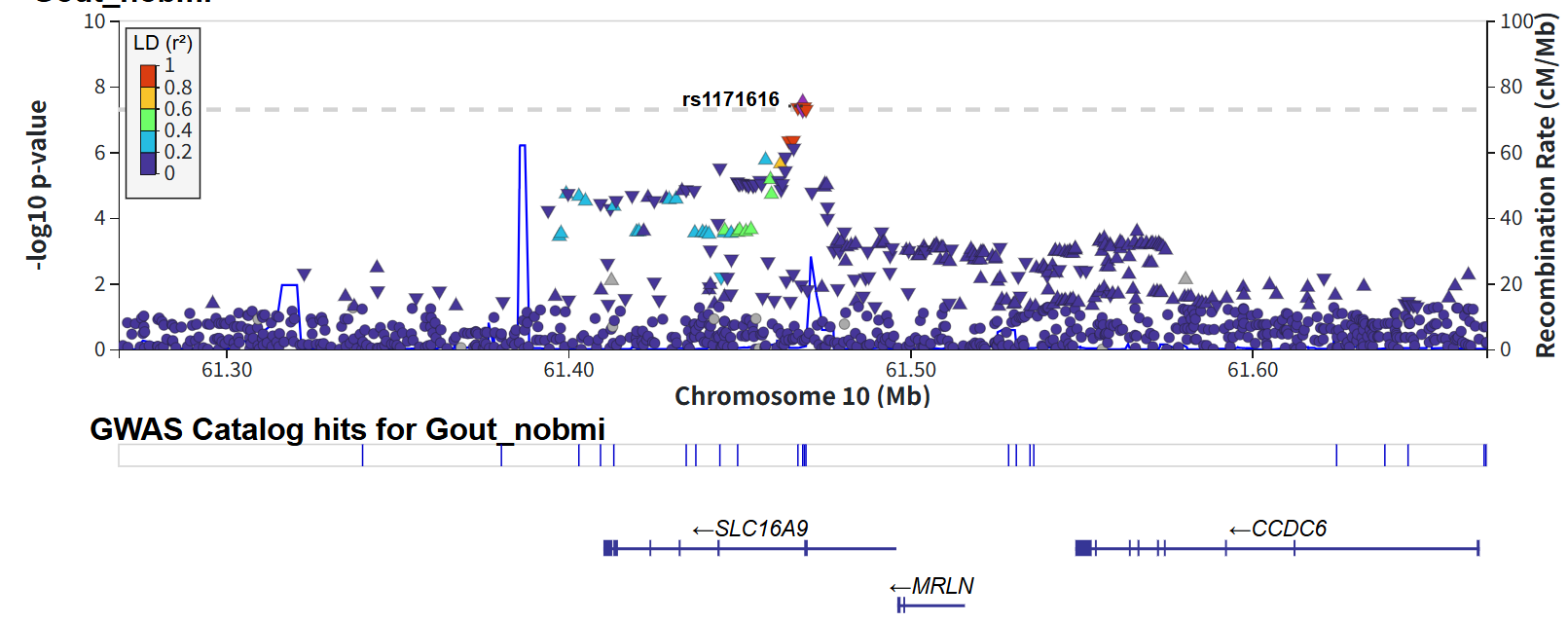
**

**Fig. S1 The regional plots of loci in the *SLC17A1*, *GCKR*, *SLC22A11*, *ADH1B*, *MLXIPL*, *RREB1* and *SLC16A9* regions from the primary GWAS.**

Panel A shows the regional plot for the *SLC17A1* locus with the lead SNP rs11279697 marked in purple. Panel B displays the regional plot for the *GCKR* locus with the lead SNP rs1260326 marked in purple. Panel C shows the regional plot for the *SLC22A11* locus with the lead SNP rs17300741 marked in purple. Panel D displays the regional plot for the *ADH1B* locus with the lead SNP rs1229984 marked in purple. Panel E shows the regional plot for the *MLXIPL* locus with the lead SNP rs17145750 marked in purple. Panel F displays the regional plot for the *RREB1* locus with the lead SNP rs13206608 marked in purple. Panel G shows the regional plot for the *SLC16A9* locus with the lead SNP rs1171616 marked in purple. Each plot illustrates SNP associations in the respective regions from the primary GWAS analysis. The x-axis represents the genomic position in Mb, while the y-axis displays the association strength of each SNP as –log^10^ *p* value.


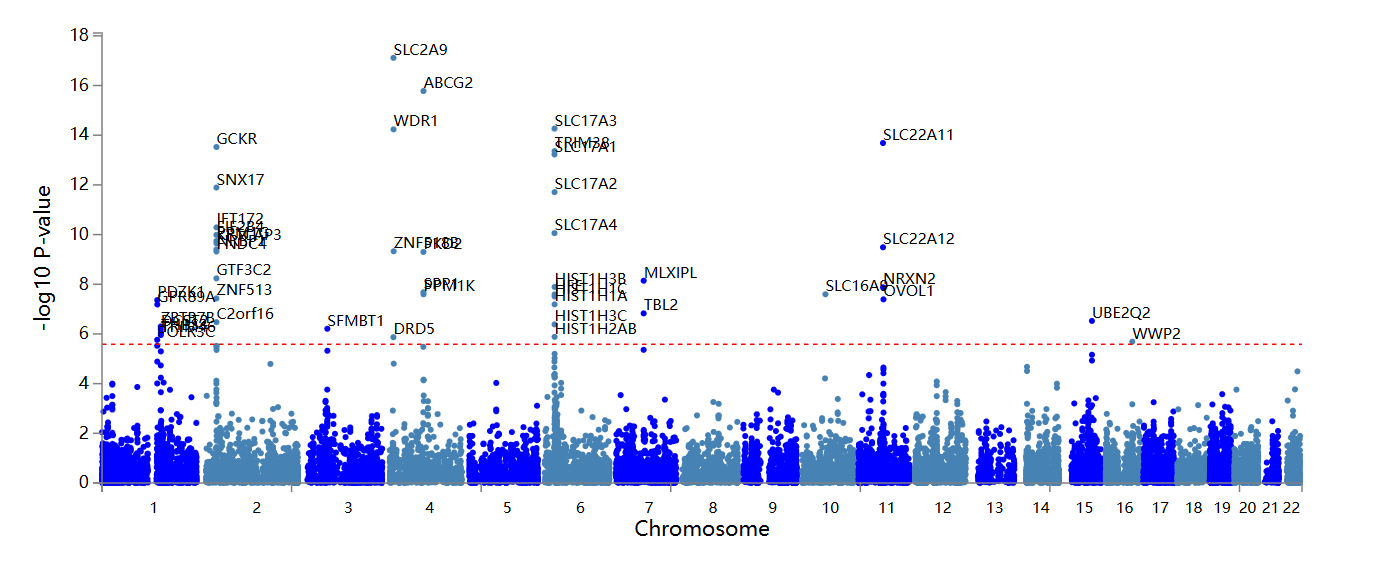


**Fig. S2 Manhattan plot of gene-based associations computed by MAGMA for gout**

The Manhattan plot displays the results of a gene-based test computed by MAGMA using GWAS summary statistics. Each dot represents a protein-coding gene mapped from the input SNPs (N = 19,203 genes). The y-axis shows the -log^10^(*p* value) of gene-based associations, while the x-axis represents genomic positions across chromosomes. The red dashed line denotes the genome-wide significance threshold (*p* = 0.05/19,203 = 2.604 × 10^-6^). Genes that surpass this threshold are labeled in the plot, indicating their significant association with knee pain.


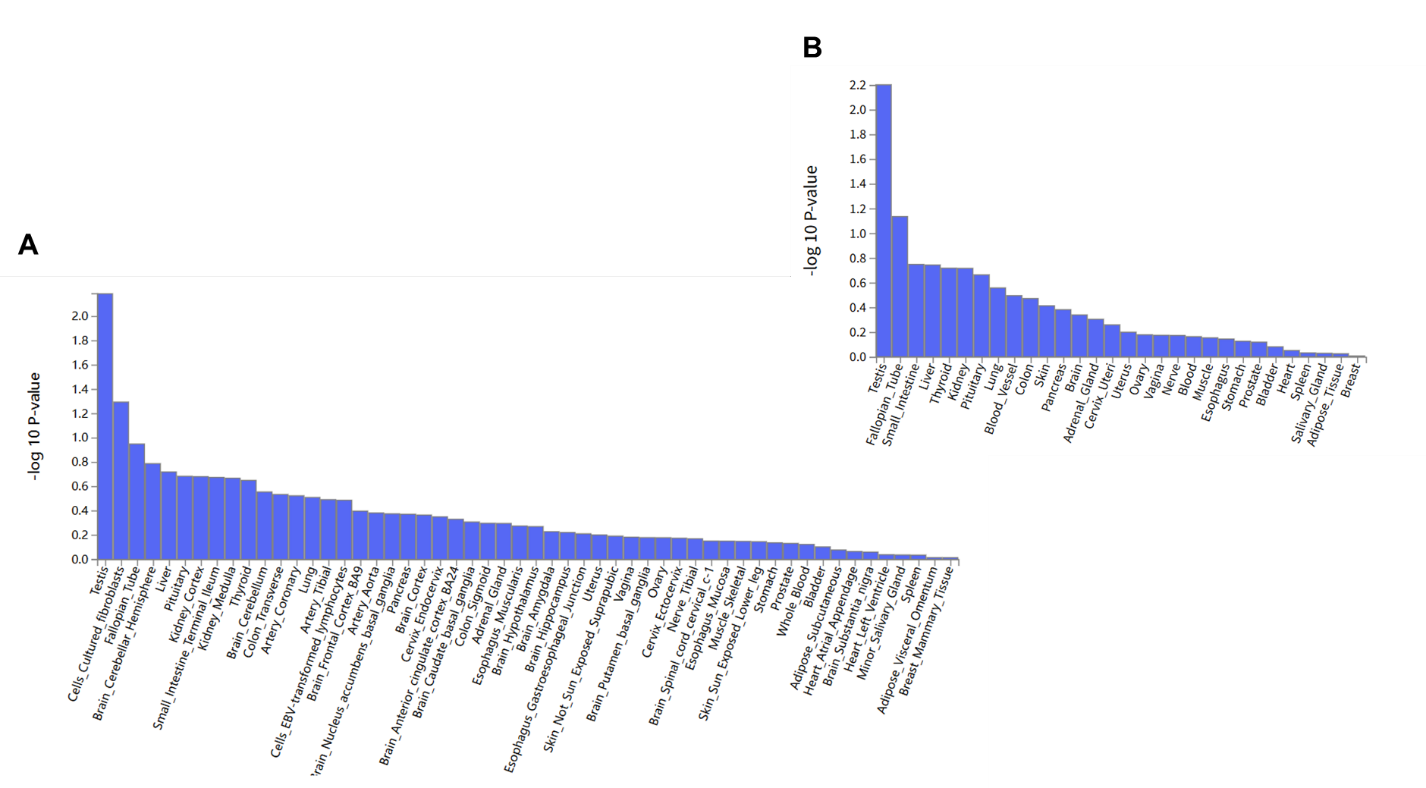


**Fig. S3 Tissue expression results for 30 and 53 specific tissue types from GTEx using FUMA.**

Panel A shows tissue expression results for 53 specific tissue types from GTEx, analyzed with FUMA. Panel B displays results for 30 tissue types. In both panels, the dashed line indicates the significance threshold, adjusted for multiple comparisons using Bonferroni correction. Significant tissue associations are highlighted in red.


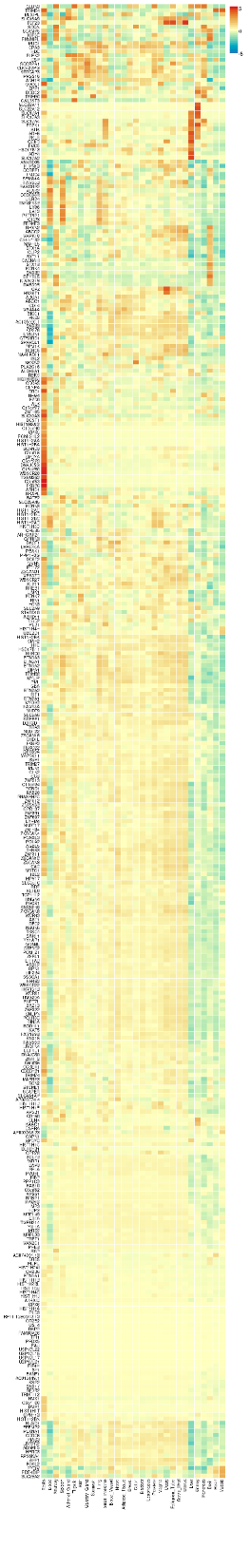

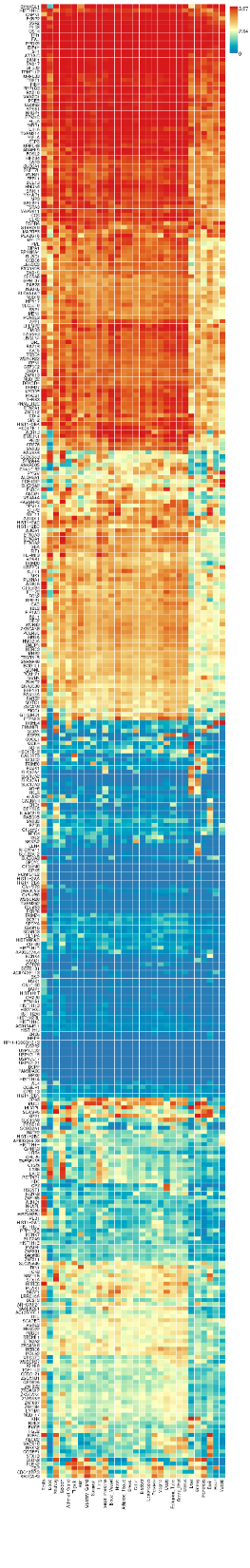

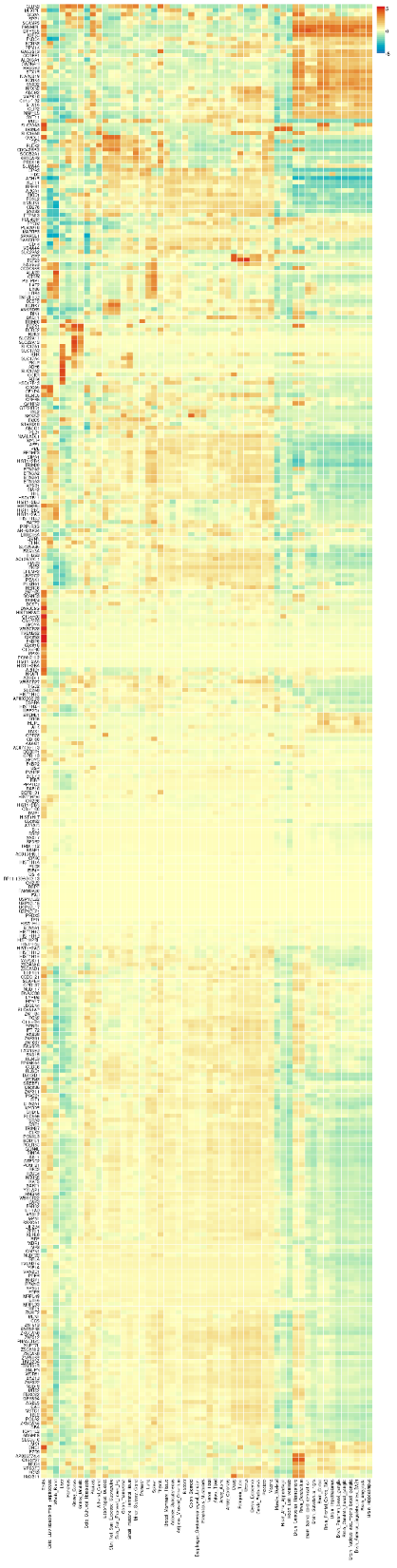

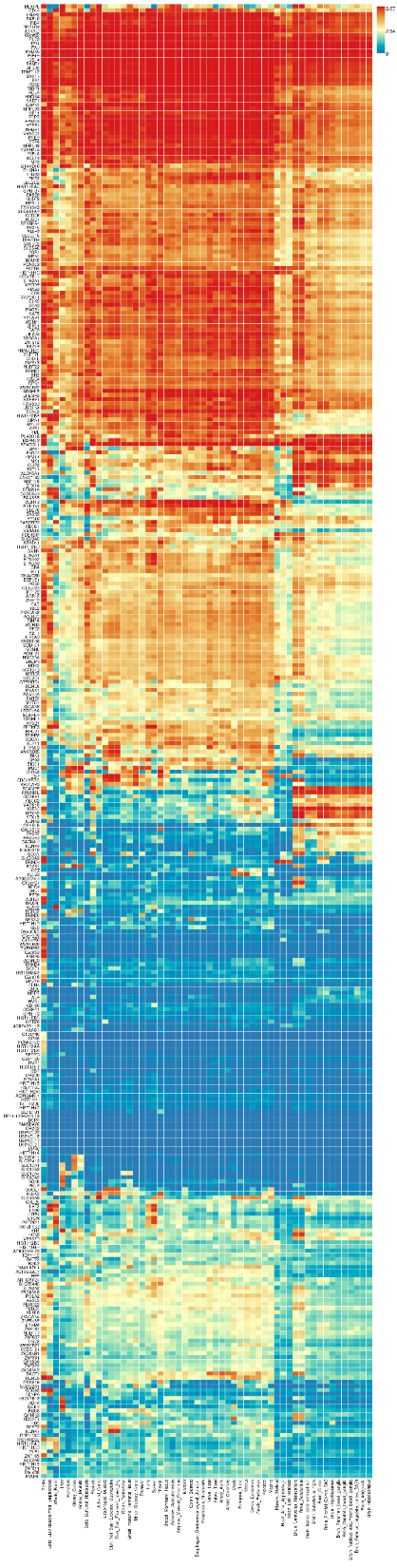


C

D

B

A

**Fig. S4 Heatmaps of gene expression across 54 specific and 30 general tissue types from GTEx v8.**

The four heatmaps visualize gene expression values for 54 specific tissue types and 30 general tissue types from GTEx v8, with genes represented as rows and tissues as columns. Panel A shows the average expression per label for 54 specific tissue types (log2 transformed). Panel B represents the average of normalized expression per label (zero mean across samples) for 54 specific tissue types. Panel C displays the average expression per label for 30 general tissue types (log2 transformed). Panel D shows the average of normalized expression per label (zero mean across samples) for 30 general tissue types. Red cells indicate higher expression relative to other tissues and genes, while blue cells indicate lower expression.


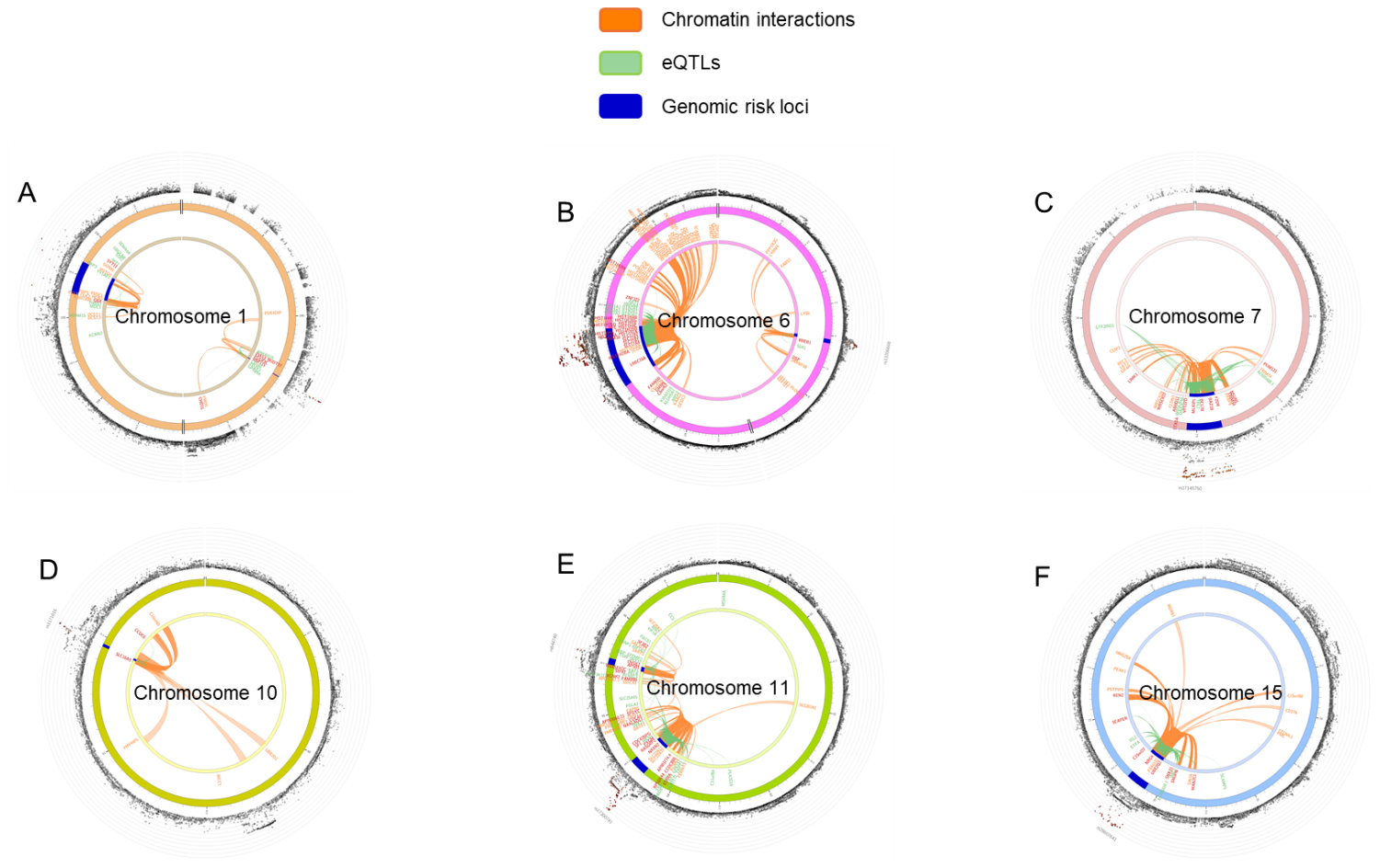


**Fig. S5 Circos plot illustrating chromatin interactions and eQTL**

The Circos plot displays chromatin interactions and eQTL associated with genomic risk loci. The outermost layer shows a Manhattan plot with loci meeting the significance threshold of *p* < 0.05. Each SNP in a genomic risk locus is color-coded according to its maximum LD, measured by r² with an independent significant SNP in the locus: red (r² > 0.8), orange (r² > 0.6), green (r² > 0.4), and blue (r² > 0.2). The middle layer marks genomic risk loci (with lead SNP *p* < 5 × 10-8) in blue. The innermost layer highlights eQTLs (green) and/or chromatin interactions (orange). Panels A to F show specific top SNPs on chromosomes 1, 6, 7, 10, 11and 15, respectively.


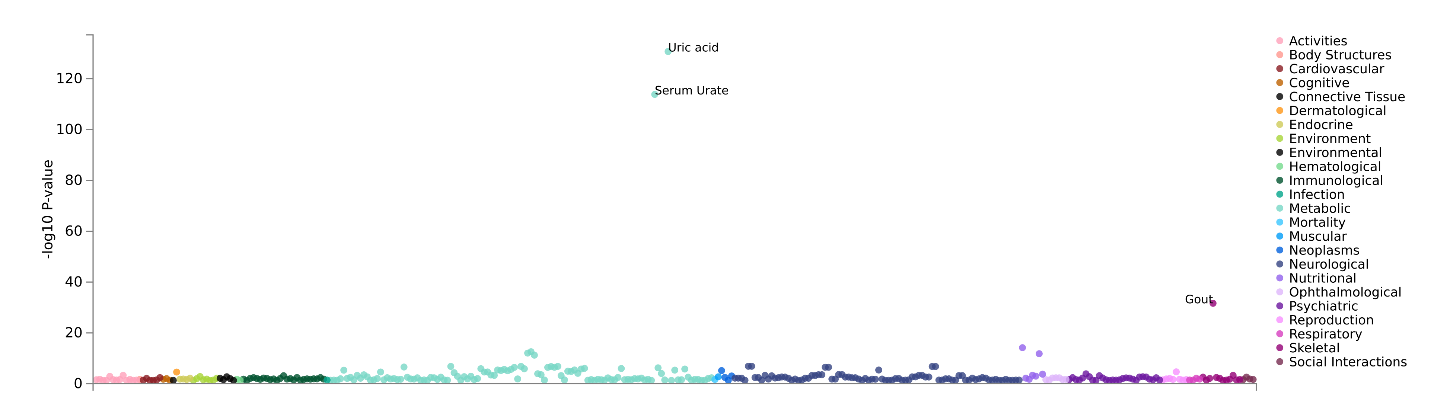


A


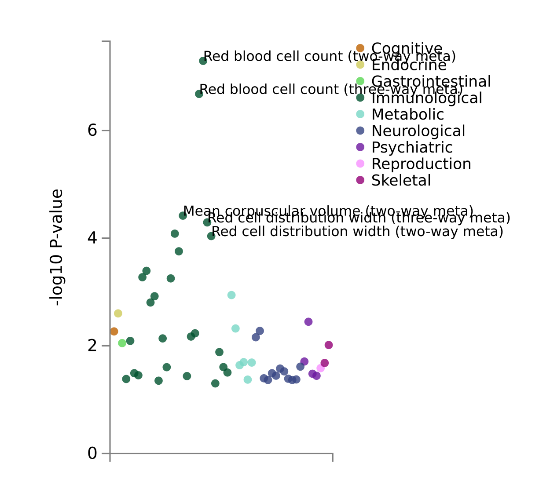


C

B


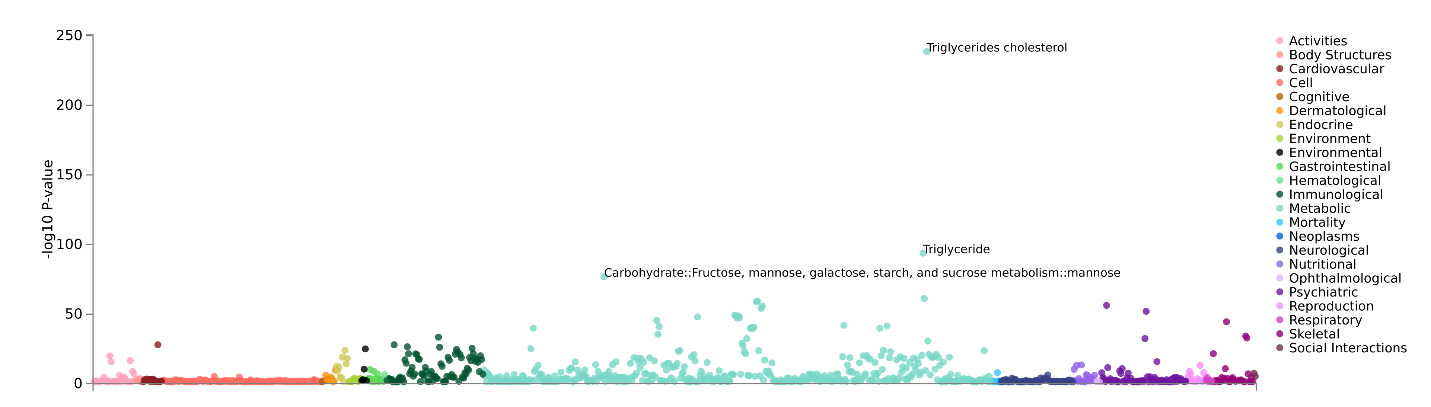


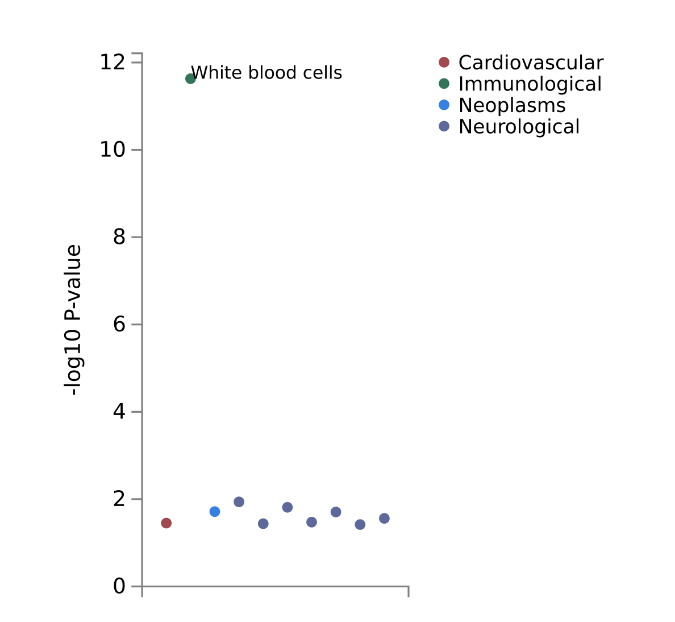

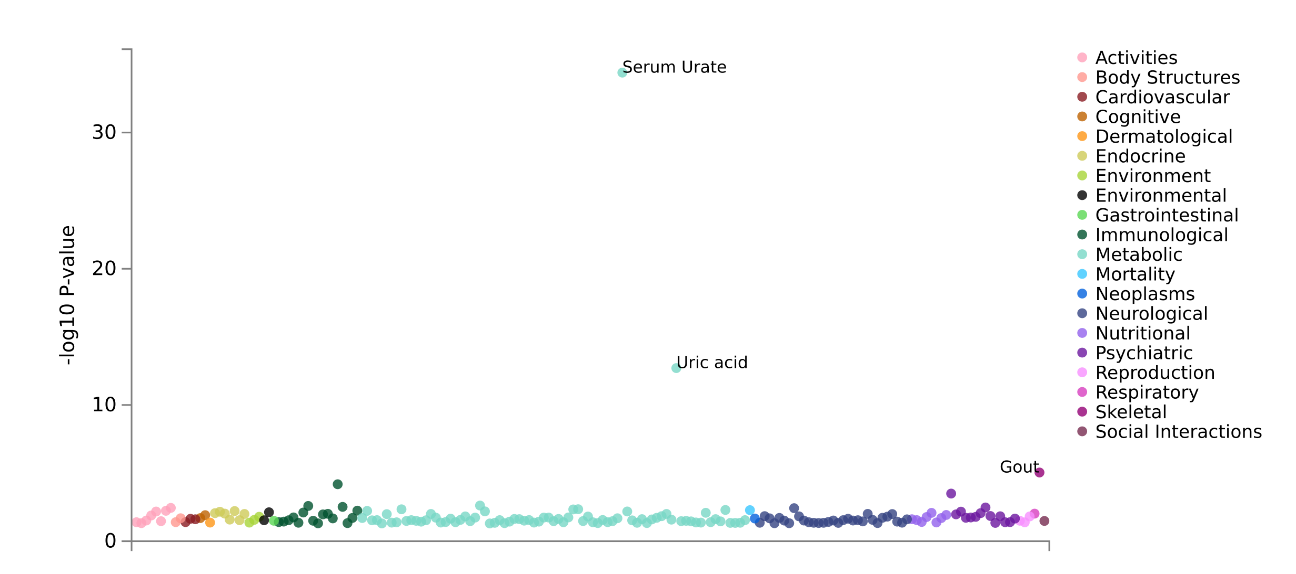

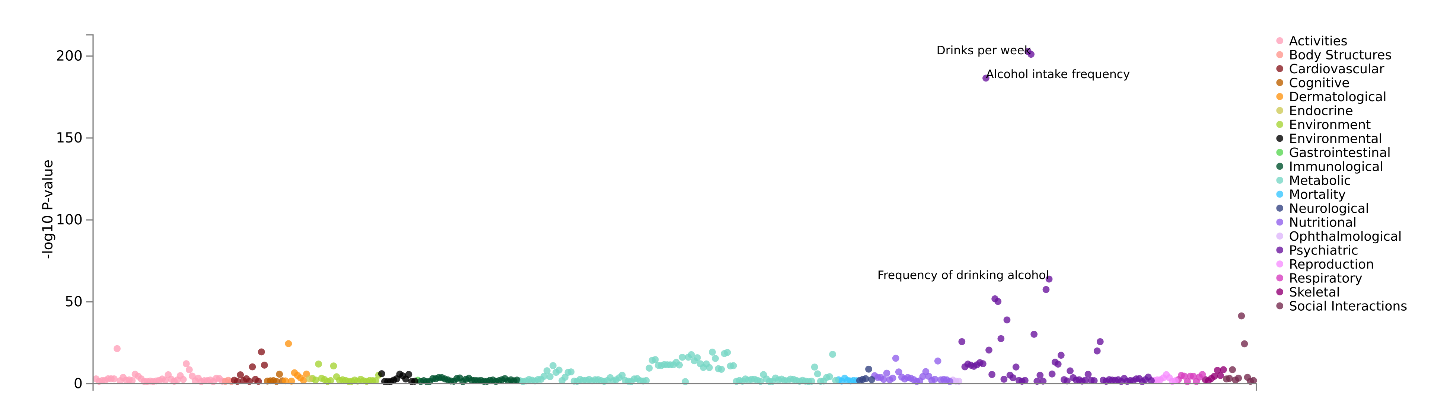


F

E

D


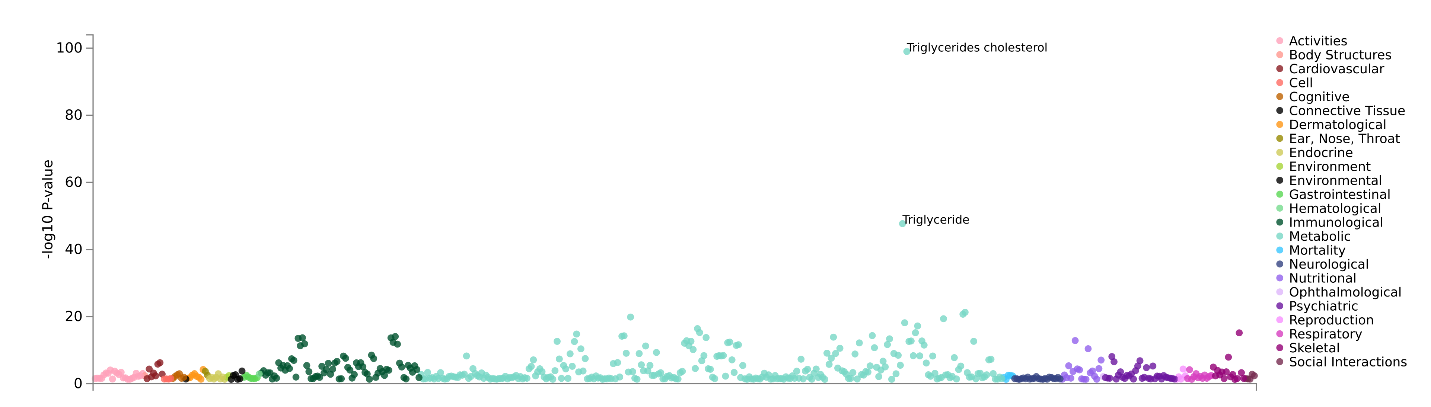

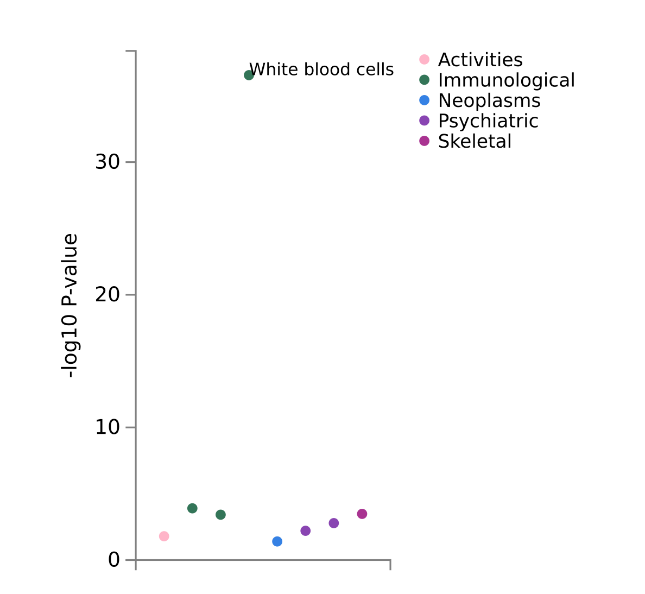

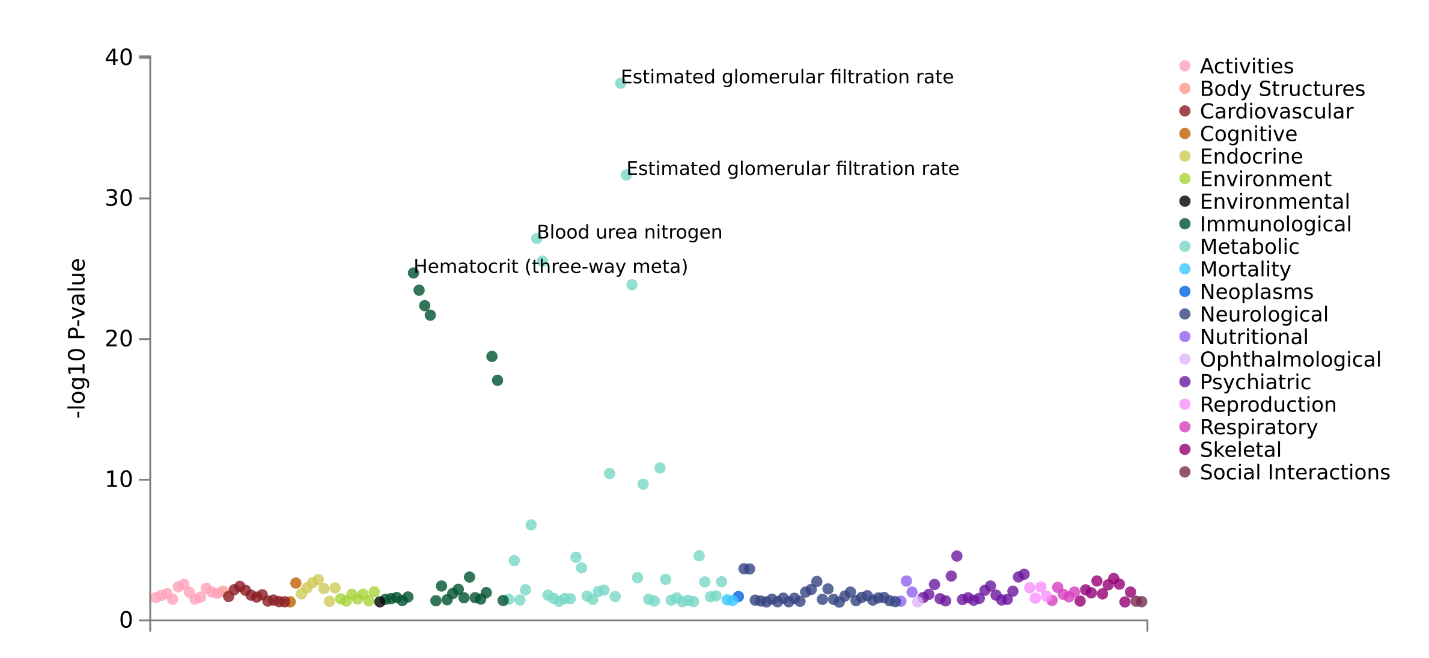


I

H

G


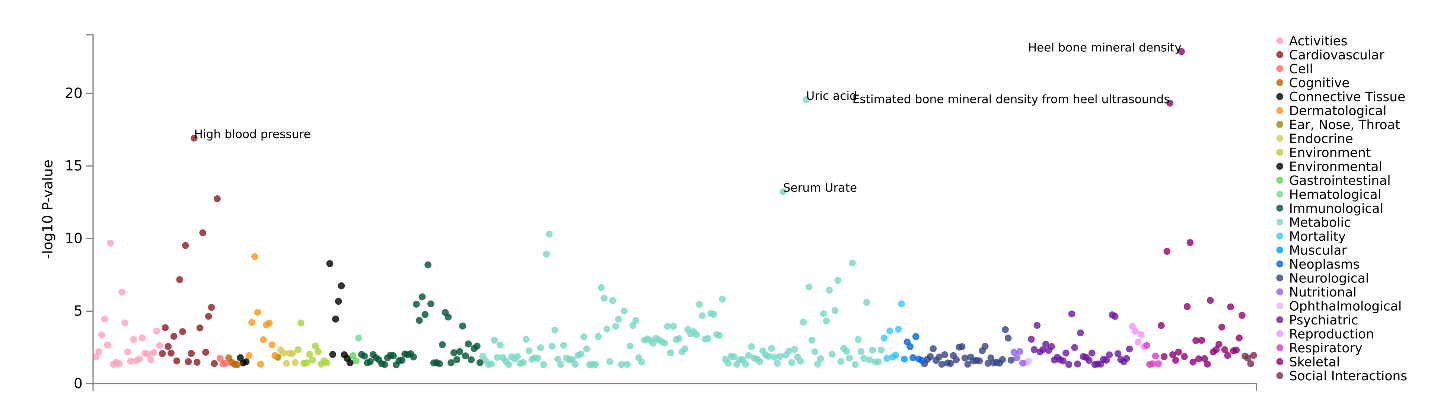

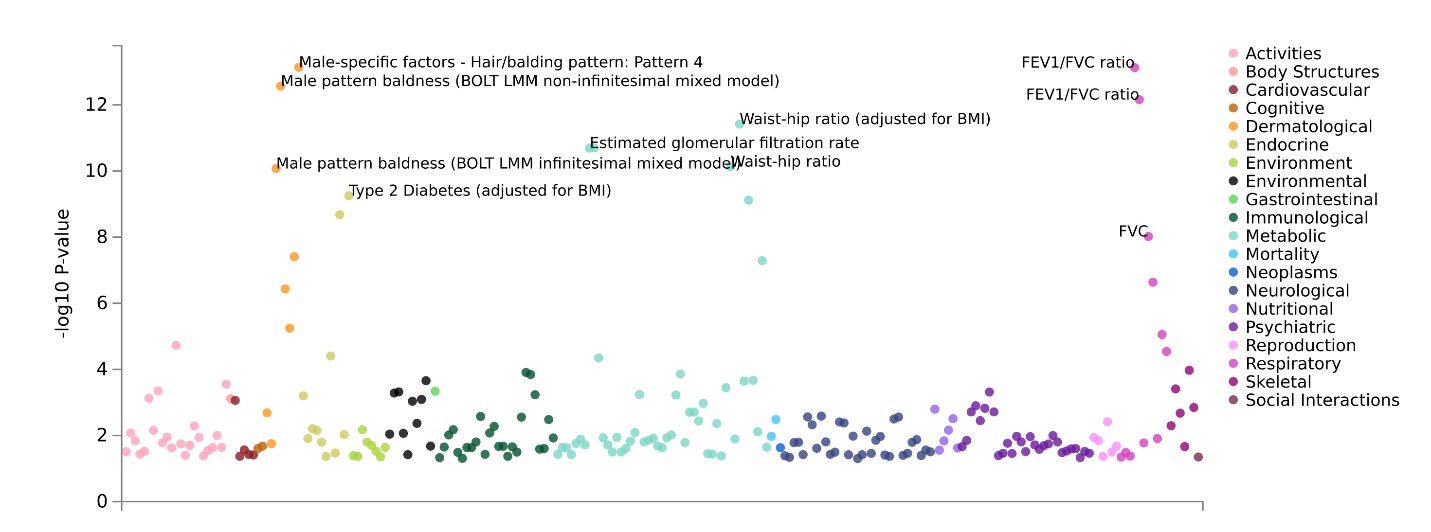

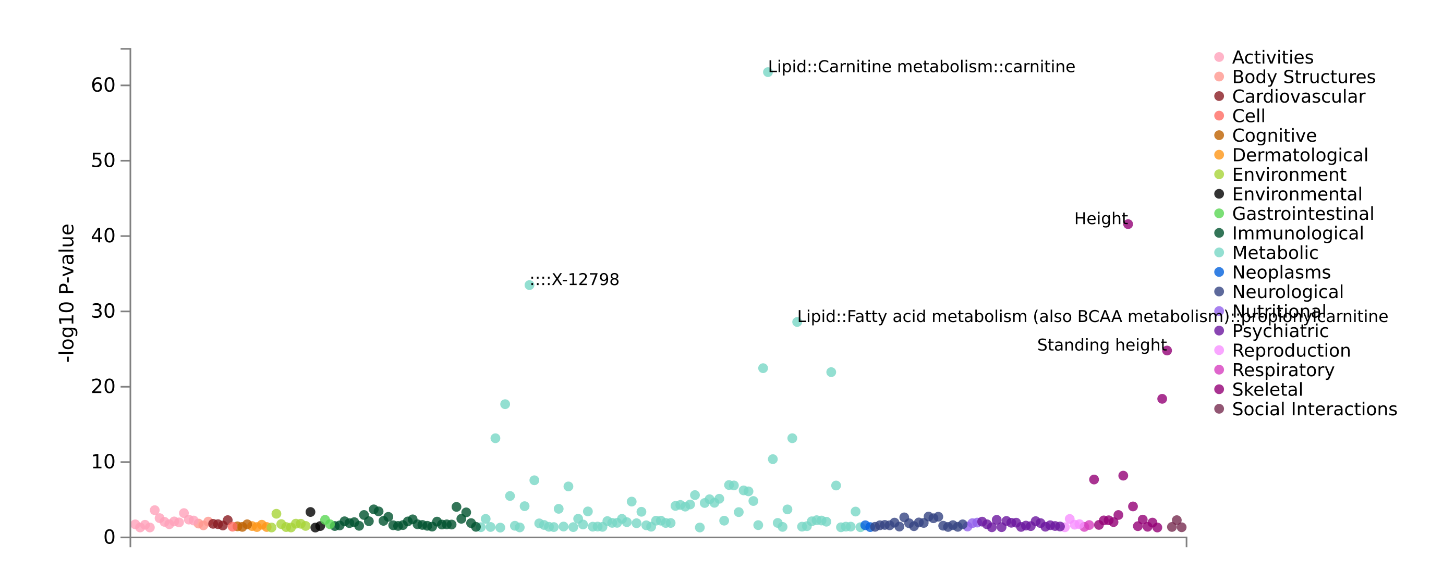


L

K

J

**Fig. S6 Phenome-wide associations with significant SNPs for gout.**

This plot illustrates the phenome-wide associations of significant SNPs linked to knee pain across various phenotypic categories. Each panel represents the associations for a specific SNP: Panel A shows results for rs2199936, Panel B for rs58656183, Panel C for rs1260326, Panel D for rs17300741, Panel E for rs1229984, Panel F for rs149865899, Panel G for rs28607641, Panel H for rs3041216, Panel I for rs17145750, Panel J for rs13206608, Panel K for rs644740 and Panel L for rs1171616. Each dot represents a phenotype association, with the x-axis showing phenotype categories and the y-axis displaying the -log^10^*p* value) for each association. Phenotypes are color-coded according to broader phenotype categories. Significant associations are labeled for clarity.

A


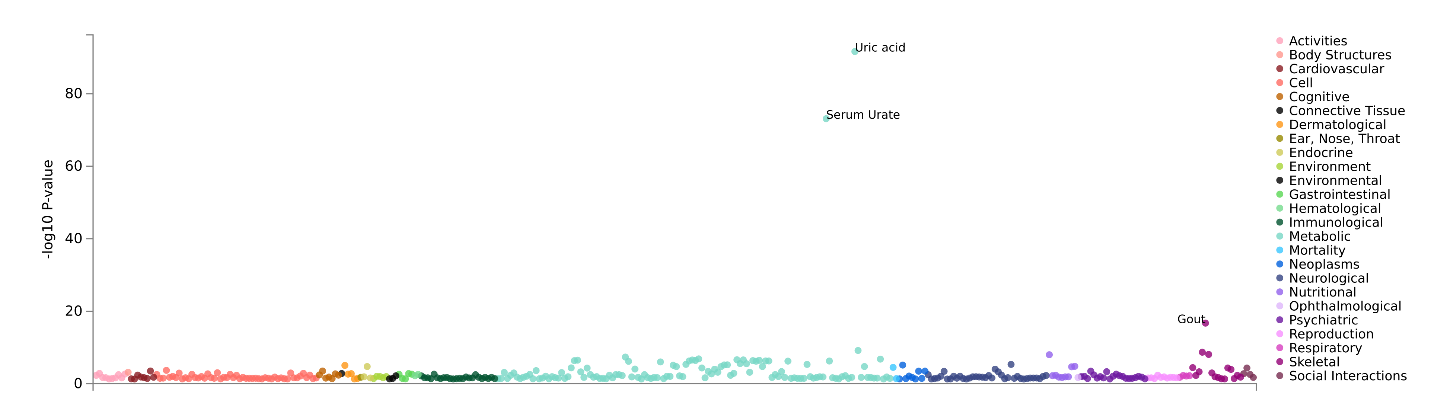


B


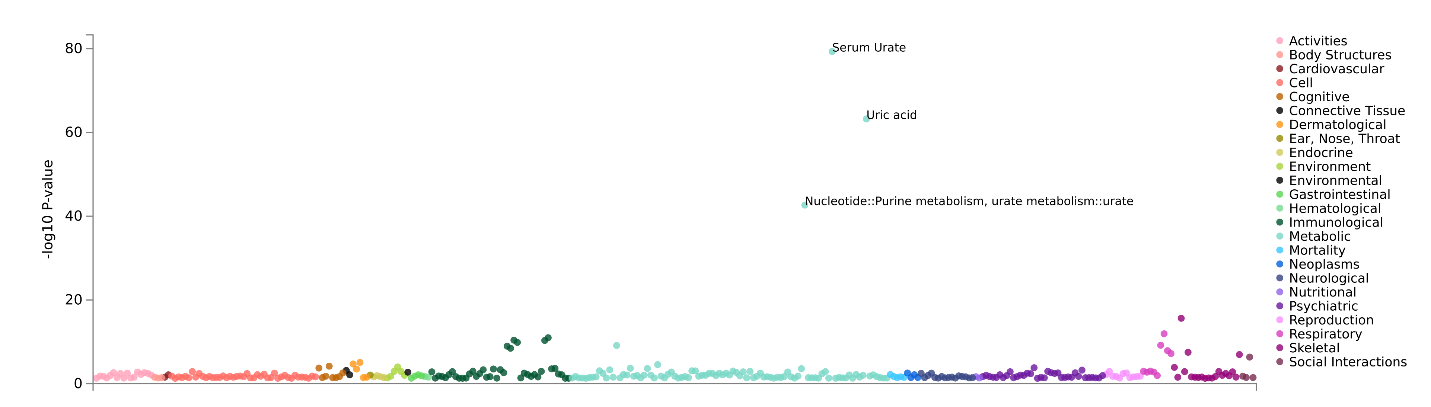


**
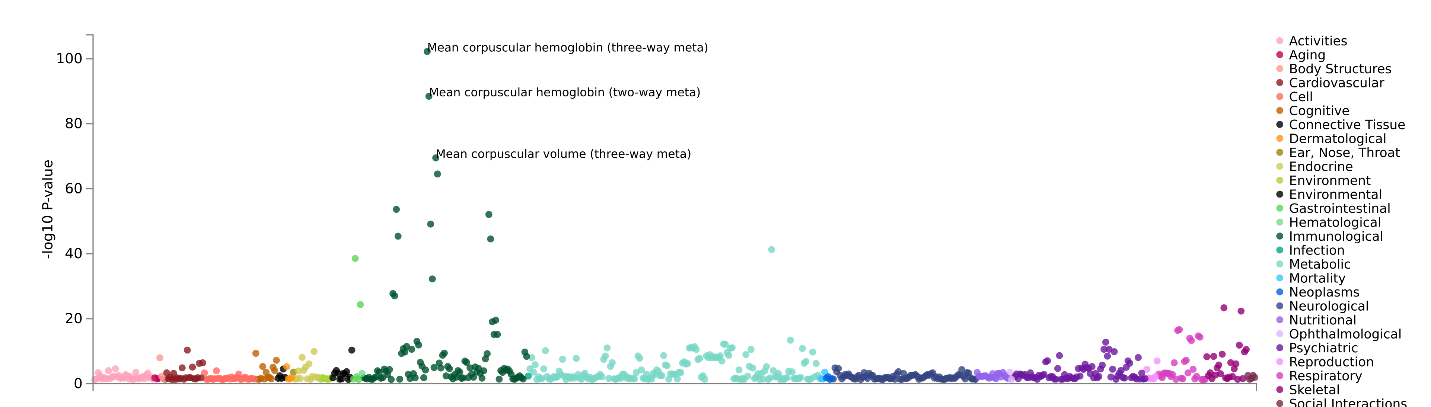
**

D

C

**
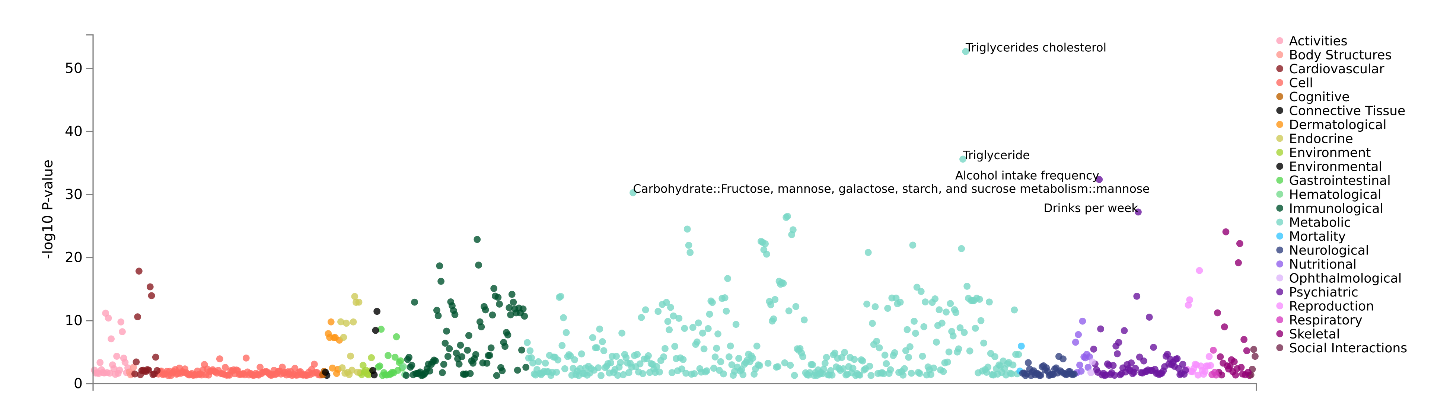
**

E

**
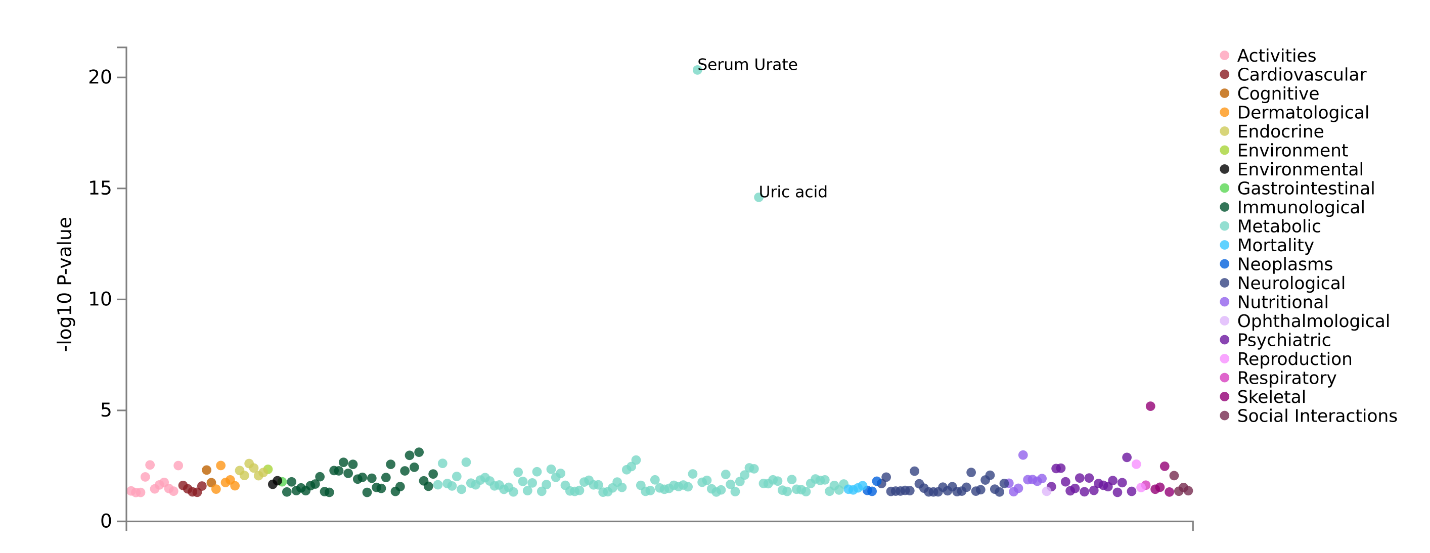
**

F

**
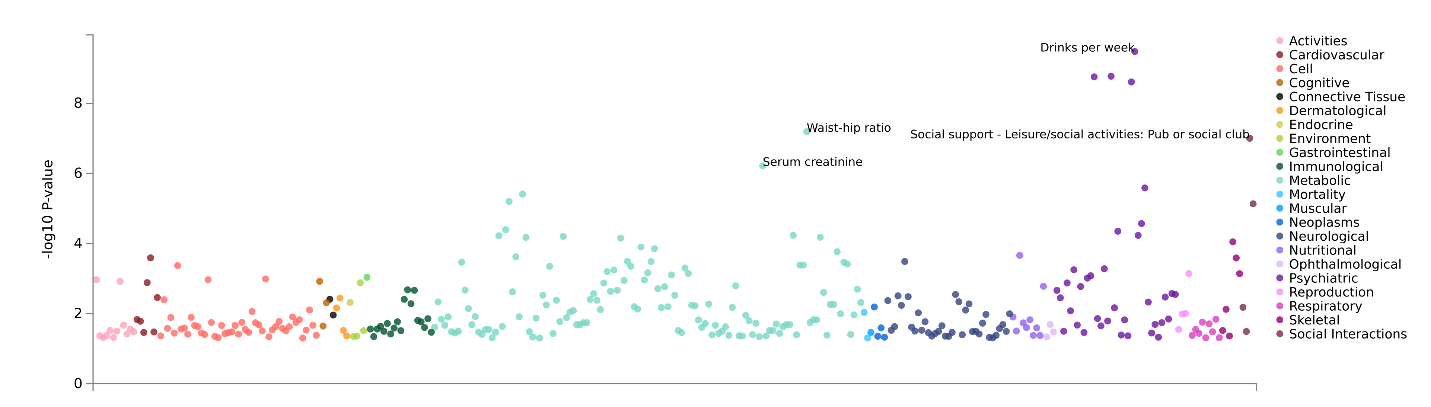

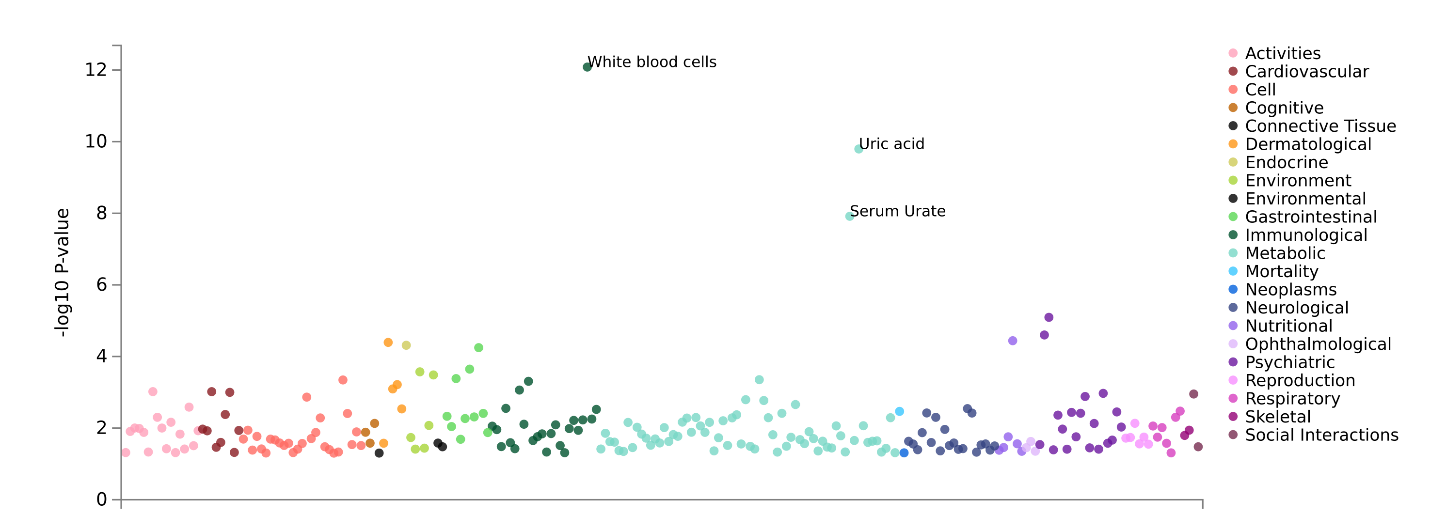

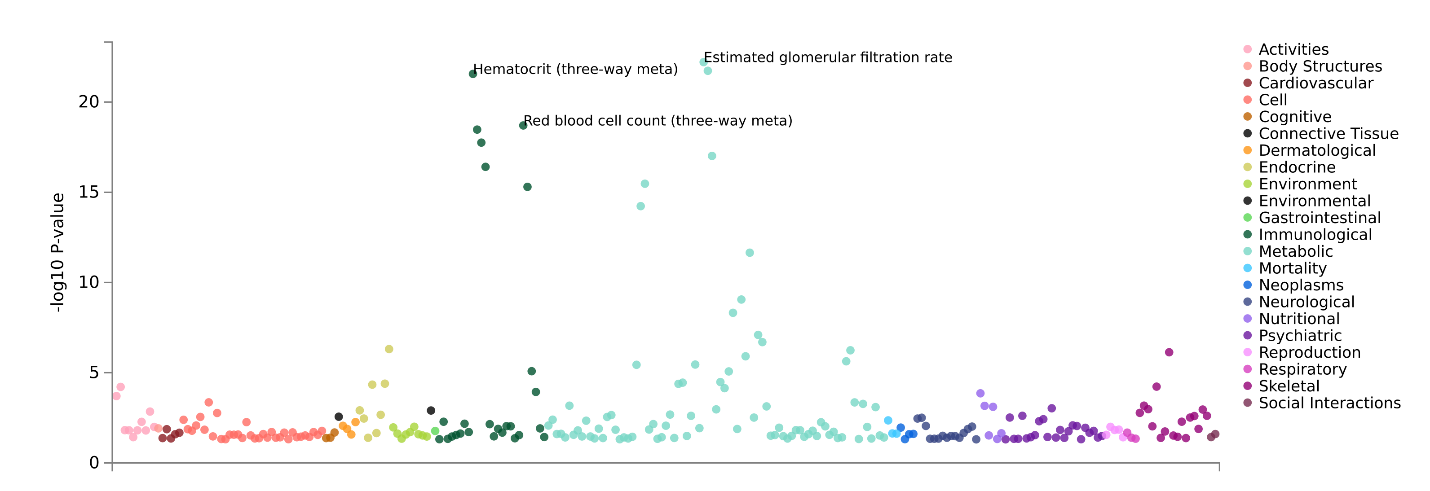

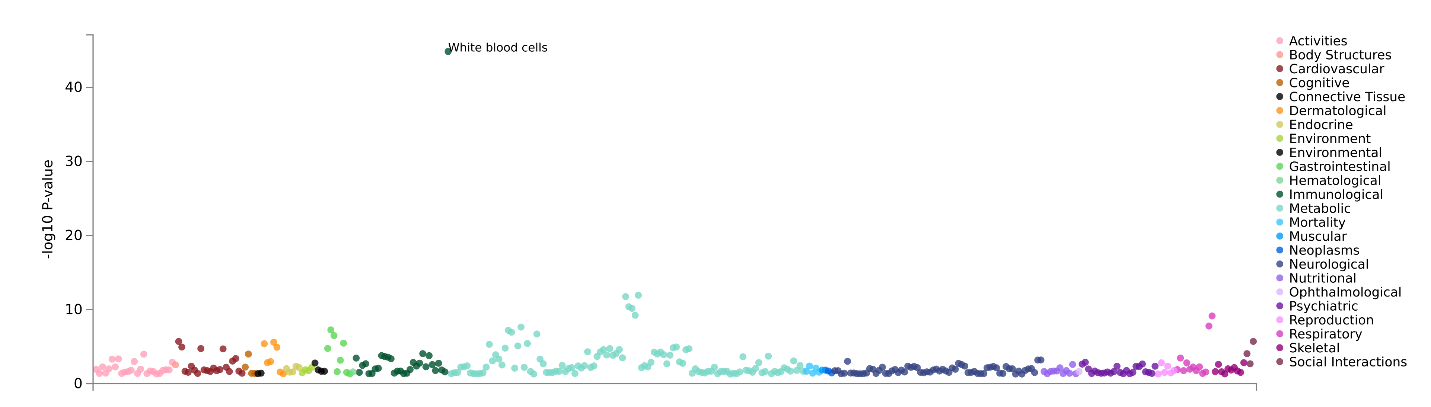
**

H

G

J

I

**
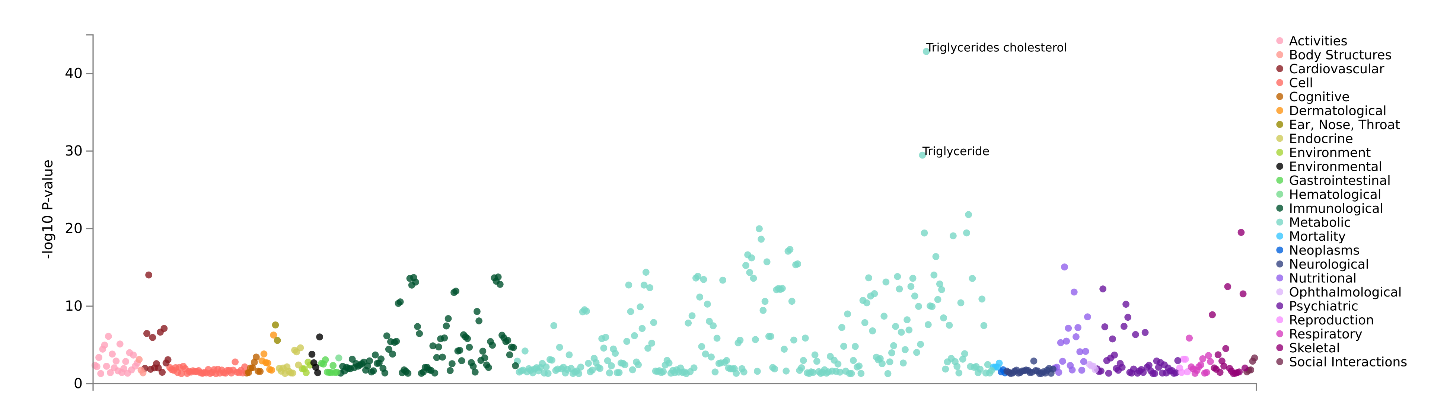

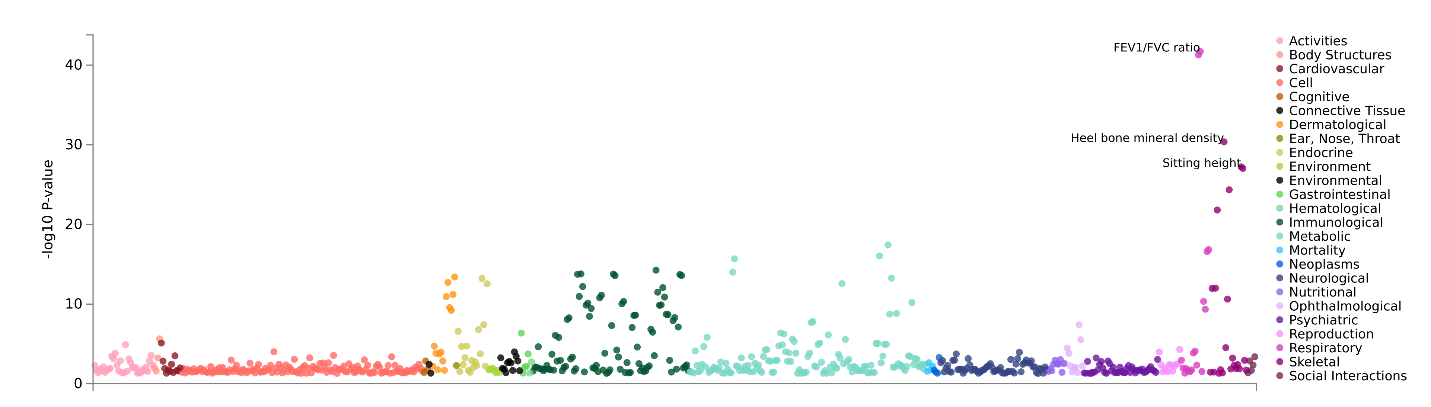

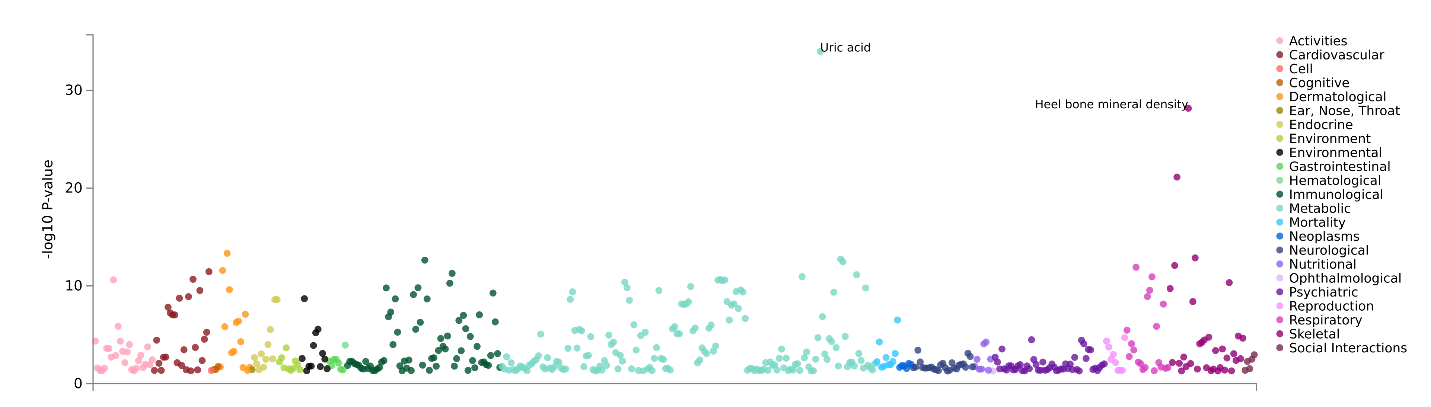

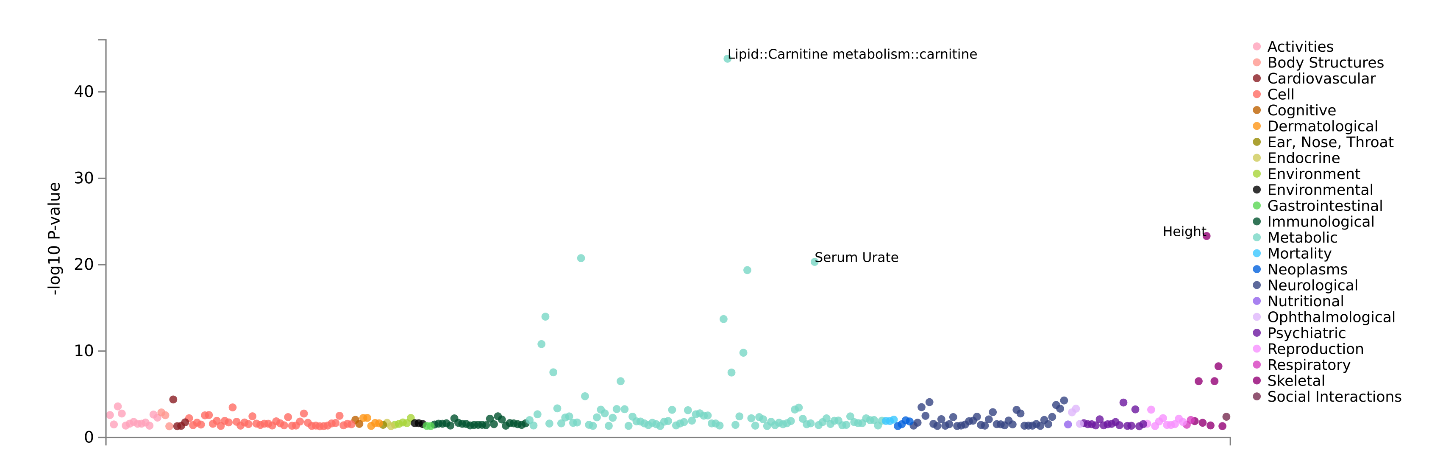
**

L

K

M

**Fig. S7 Phenome-wide associations with significant genes for gout.**

This plot illustrates the phenome-wide associations of significant genes linked to knee pain across various phenotypic categories. Each panel represents the associations for a gene: Panel A shows results for *ABCG2*, Panel B for *SLC2A9*, Panel C for *SLC17A1*, Panel D for *GCKR*, Panel E for *SLC22A11*, Panel F for *ADH1B*, Panel G for *CD160*, Panel H for *UBE2Q2*, Panel I for *DAP3*, Panel J for *MLXIPL*, Panel K for *RREB1*, Panel L for *OVOL1* and Panel M for *SLC16A9*. Each dot represents a phenotype association, with the x-axis showing phenotype categories and the y-axis displaying the -log^10^*p* value) for each association. Phenotypes are color-coded according to broader phenotype categories. Significant associations are labeled for clarity.


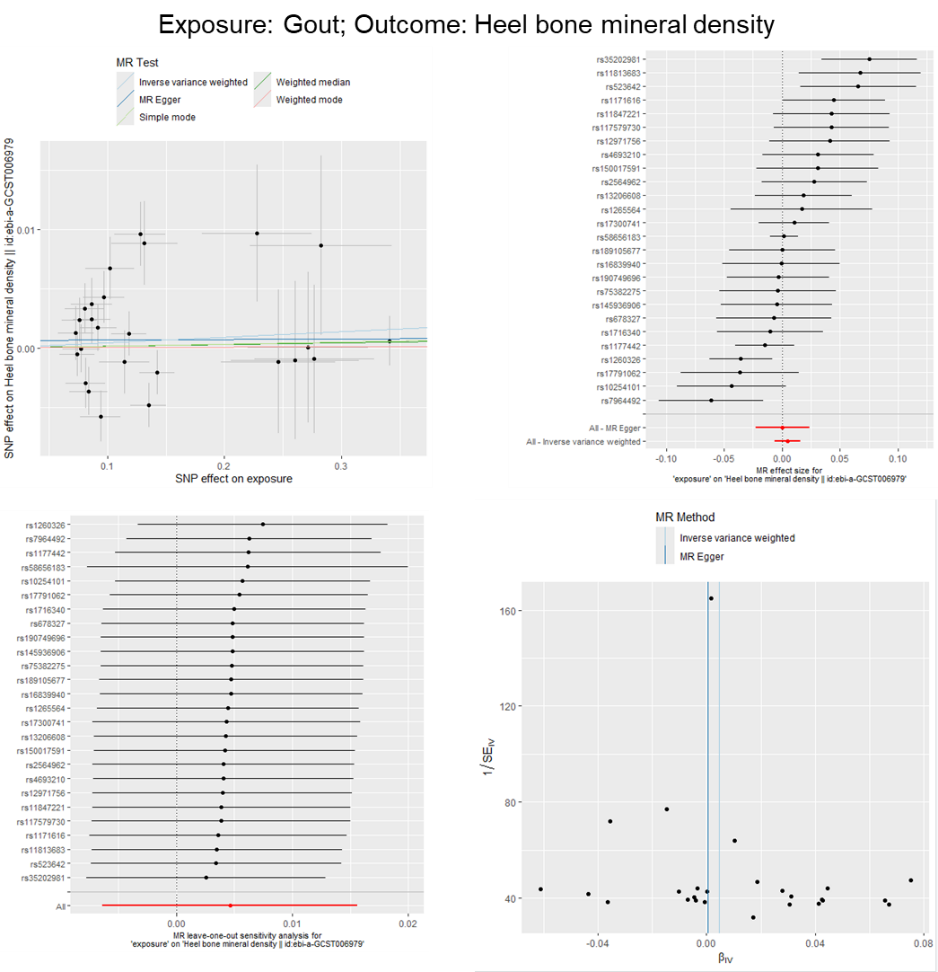

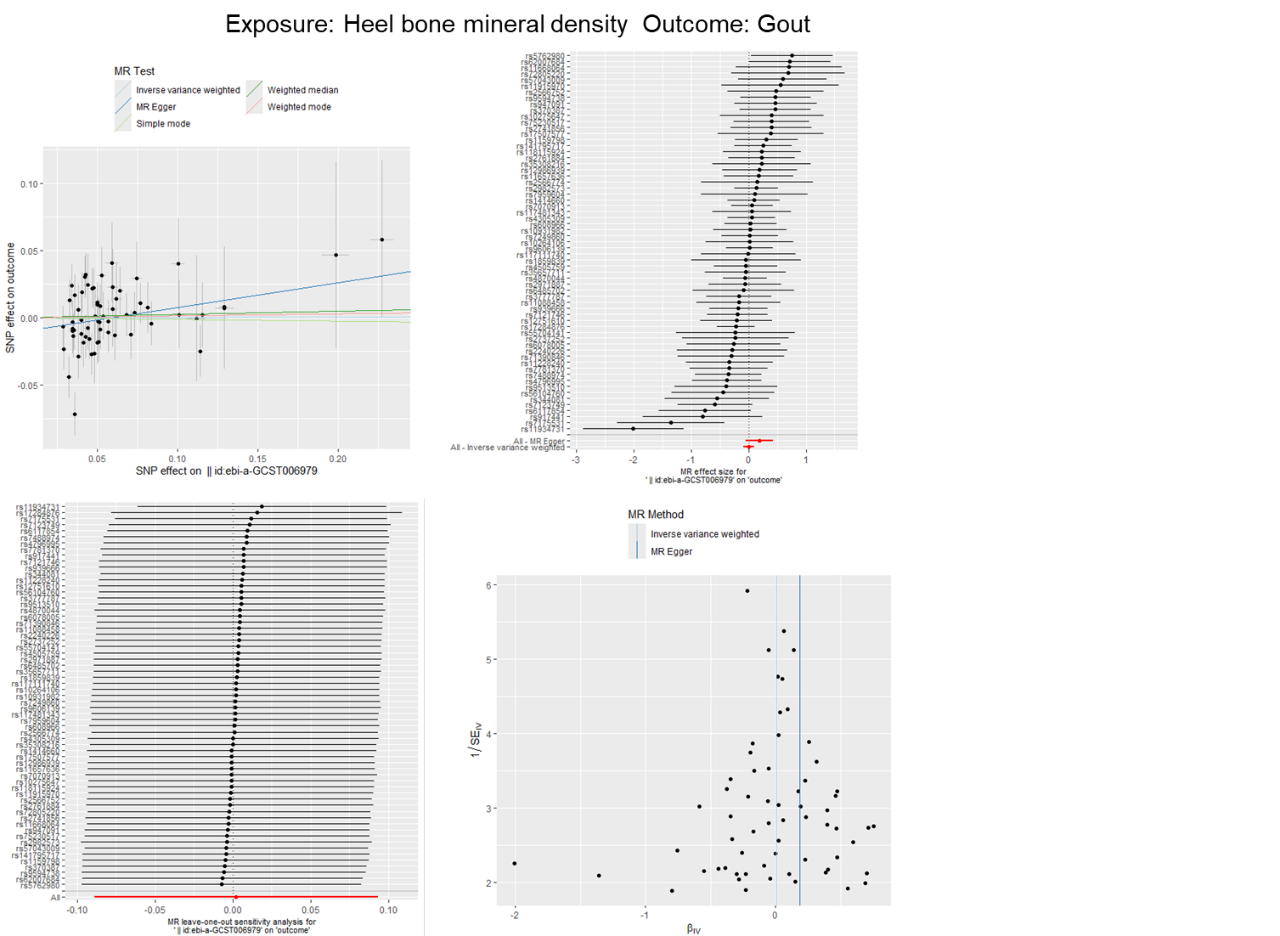


A

B


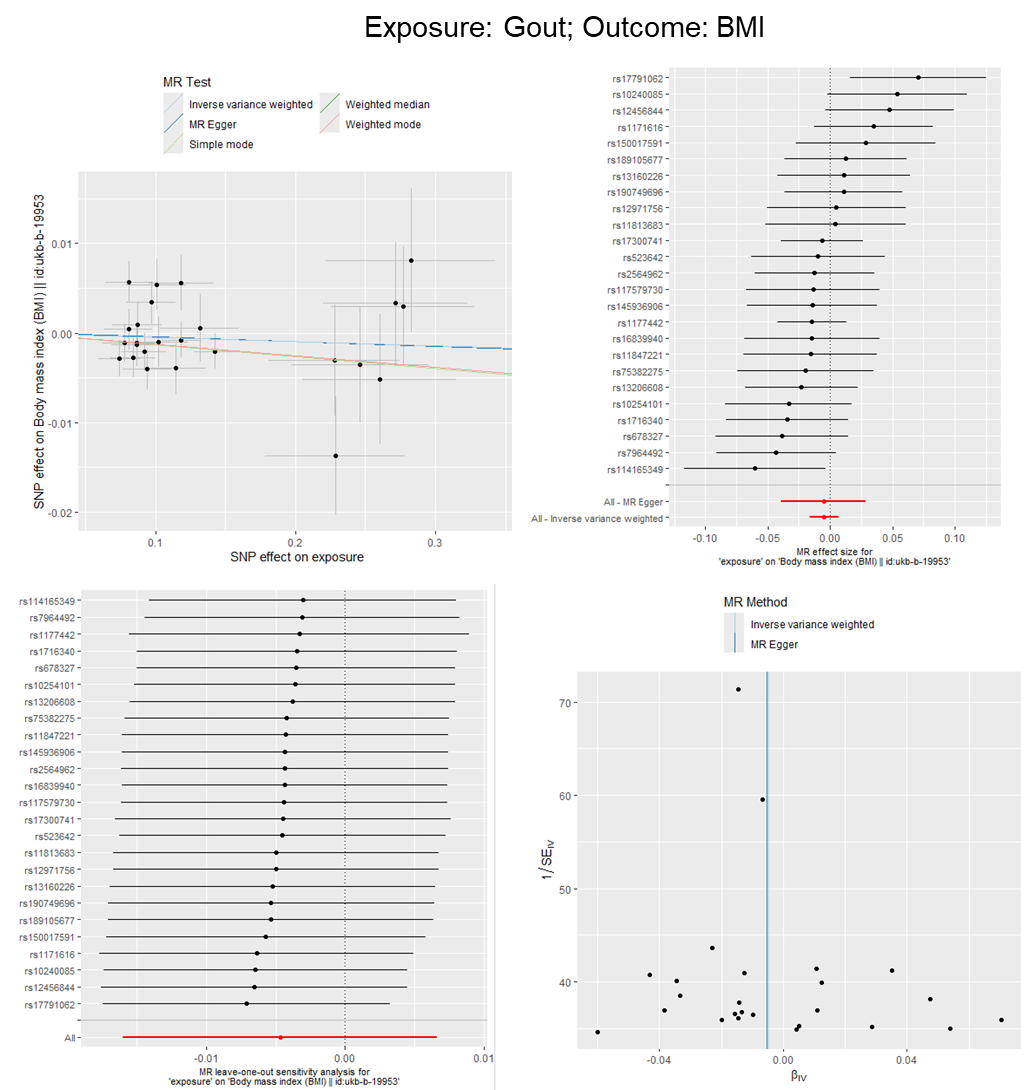

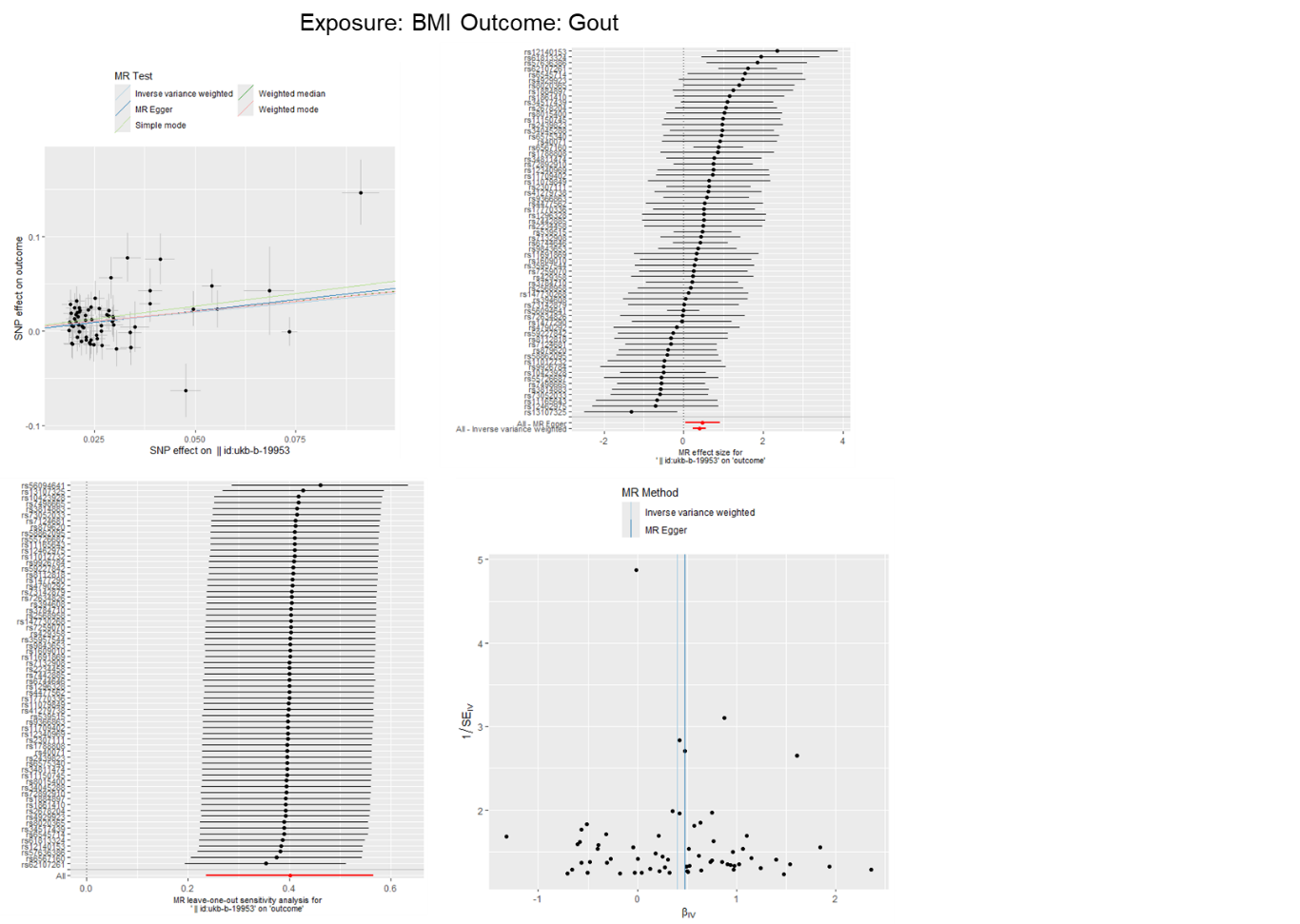


C

D


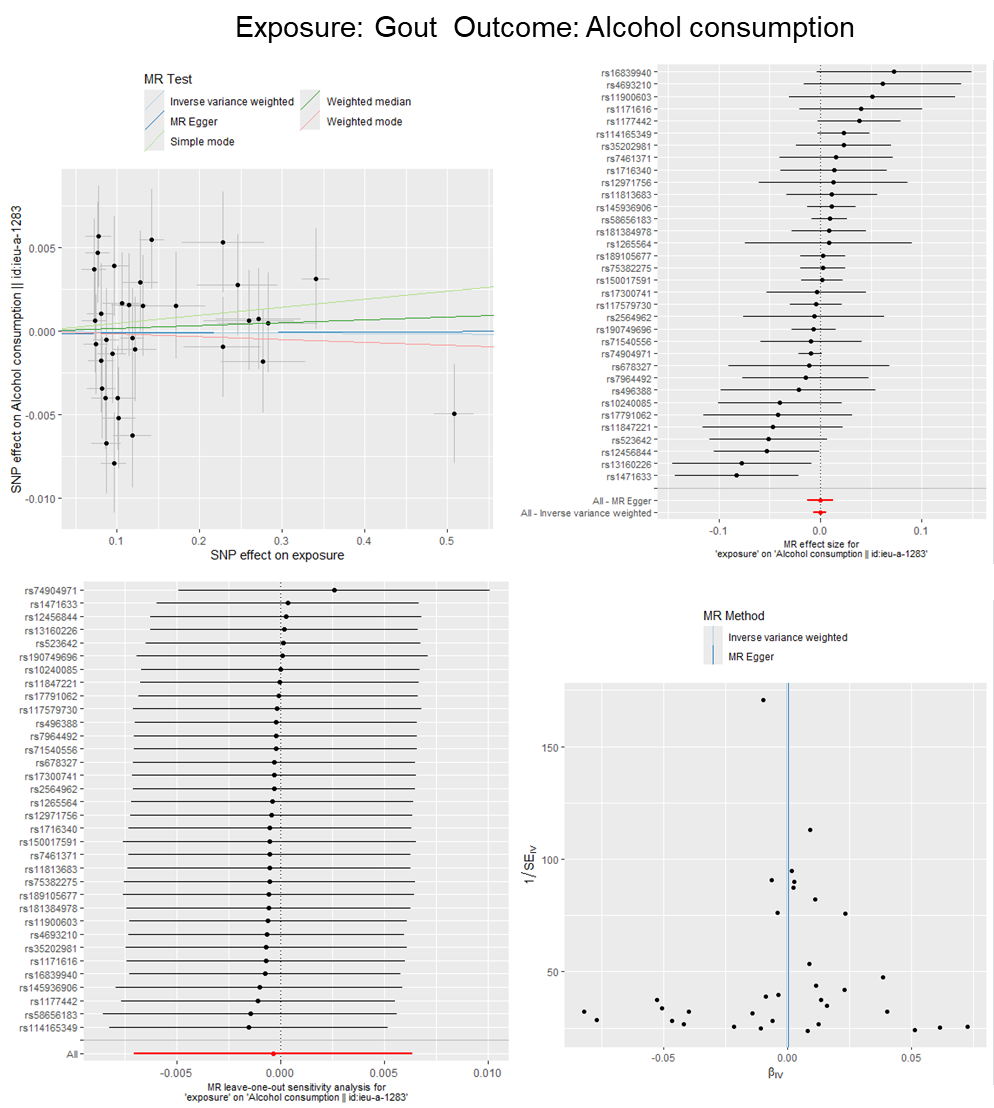

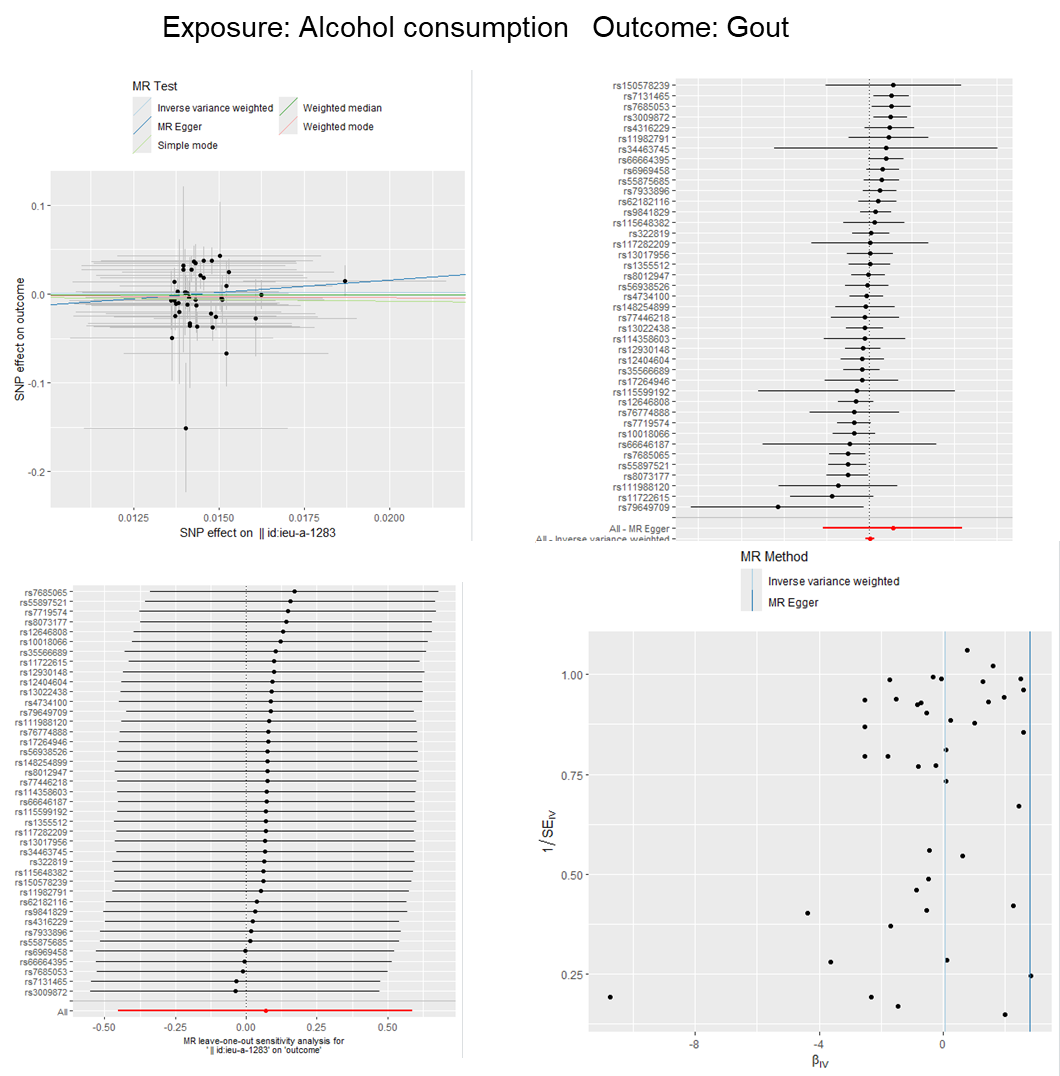


F

E

**Fig. S8 Mendelian randomization analysis of knee pain and related traits.**


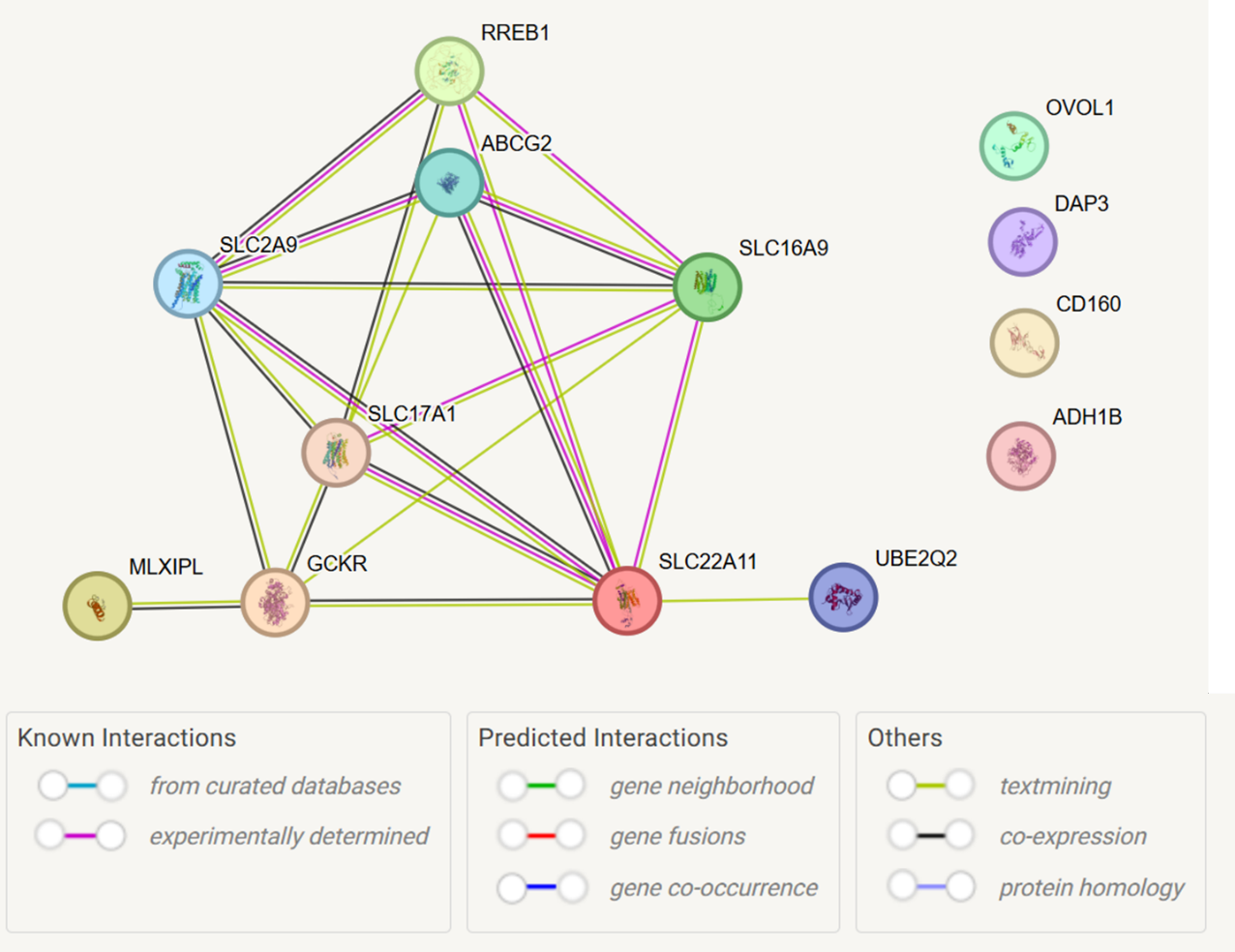
Panels A–F show MR results between gout and three musculoskeletal traits in both directions. Each panel includes four plots: a scatter plot of SNP effects on the exposure (X-axis) and their effects on the outcome (Y-axis); a forest plot displaying individual SNP effect estimates with 95% confidence intervals; a leave-one-out analysis plot evaluating the influence of each SNP on the overall estimate; and a funnel plot assessing instrument heterogeneity. Panel A presents the MR analysis from gout to heel bone mineral density, and Panel B shows the reverse direction. Panel C presents the MR analysis from gout to BMI, and Panel D shows the reverse direction. Panel E presents the MR analysis from gout to alcohol consumption, and Panel F shows the reverse direction.

**Fig. S9 Protein interaction network**

**
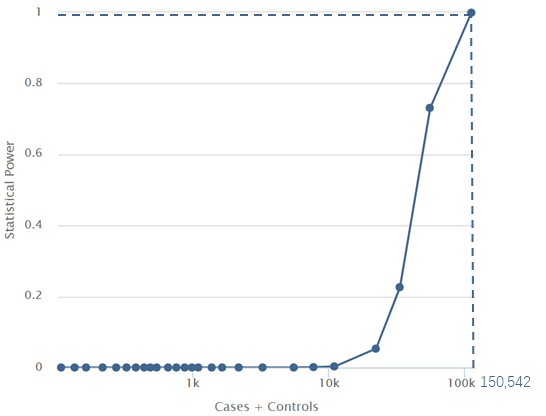

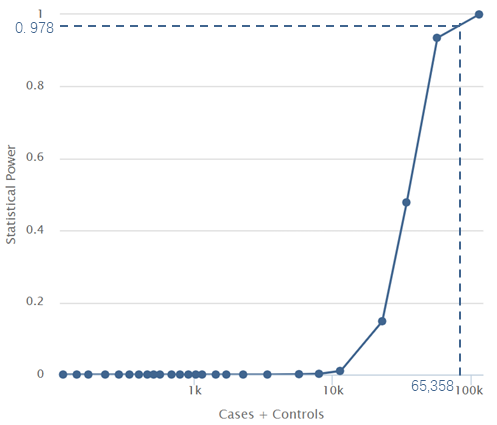
**Protein–protein interaction network constructed from the 13 significant loci identified in the primary GWAS of gout. Nodes represent proteins encoded by the associated genes, and edges indicate known or predicted interactions based on STRING database analysis.

A

B

C

**
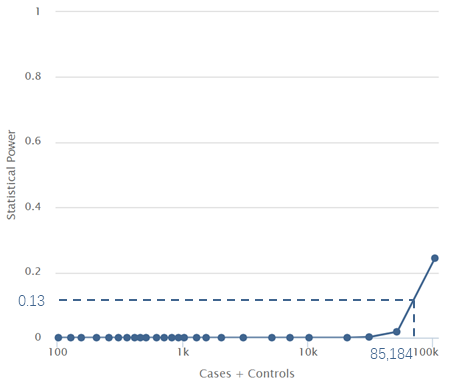
**

**Fig. S10 Power estimation for gout GWAS using the CaTS calculator.**

We used the CaTS power calculator (<http://www.sph.umich.edu/csg/abecasis/CaTS/>) to evaluate the statistical power of the primary and sex-stratified GWAS for gout. Assuming a significance level of 5 × 10^-8^, a disease prevalence of 0.0249, a disease allele frequency of 0.25, and a genotype relative risk of 1.15, the overall analysis with 10,474 cases and 140,068 controls achieved 99.9% expected power (Panel A). Sex-stratified analyses used sex-specific disease prevalence estimates: 0.0397 for males and 0.0105 for females. The male-specific GWAS (7,907 cases and 57,451 controls) also yielded full power (97.8%) (Panel B), while the female-specific GWAS (2,567 cases and 82,617 controls) had a slightly lower, but still reasonable power of 13.0% (Panel C).

**Supplementary Tables**

**Table S1. Full summary statistics of all SNPs identified in the GWAS of gout.**

**Table S2. Top ten gene sets significantly associated with knee pain from the gene set enrichment analysis.**

**Table S3. Genetic correlations between gout and a range of complex traits and conditions.**

**Table S4. Significant cis-eQTL associations between top GWAS SNPs and gene expression across tissues.**

**Table S5. Significant transcriptome-wide association study results across GTEx v7 tissue types.**

**Table S6. Significant phenome-wide association study results from GWAS Atlas platform.**

**Table S7. Bidirectional Mendelian randomization results between gout and related traits.**

**Table S8. Associations between significant genes and approved drugs.**
